# RNA-Seq transcriptome profiling in three liver regeneration models in rats: comparative analysis of partial hepatectomy, ALLPS, and PVL

**DOI:** 10.1038/s41598-020-61826-1

**Published:** 2020-03-23

**Authors:** Dilek Colak, Olfat Al-Harazi, Osama M. Mustafa, Fanwei Meng, Abdullah M. Assiri, Dipok K. Dhar, Dieter C. Broering

**Affiliations:** 1 Biostatistics, Epidemiology, and Scientific Computing Department, King Faisal Specialist Hospital and Research Center, Riyadh, Saudi Arabia; 2 Department of Surgery and Organ Transplantation Center, King Faisal Specialist Hospital and Research Center, Riyadh, Saudi Arabia; 3 Comparative Medicine Department, King Faisal Specialist Hospital and Research Center, Riyadh, Saudi Arabia; 4 Institute for Research and Medical Consultations, Imam Abdulrahman Bin Faisal University, Dammam, Saudi Arabia; 5 College of Medicine, AlFaisal University, Riyadh, Saudi Arabia; 6 Institute for Liver and Digestive Health, University College London, Royal Free Hospital, London, UK

**Keywords:** Transcriptomics, Hepatic portal vein, Translational research

## Abstract

The liver is a unique organ that has a phenomenal capacity to regenerate after injury. Different surgical procedures, including partial hepatectomy (PH), intraoperative portal vein ligation (PVL), and associated liver partition and portal vein ligation for staged hepatectomy (ALPPS) show clinically distinct recovery patterns and regeneration. The observable clinical differences likely mirror some underlying variations in the patterns of gene activation and regeneration pathways. In this study, we provided a comprehensive comparative transcriptomic analysis of gene regulation in regenerating rat livers temporally spaced at 24 h and 96 h after PH, PVL, and ALPPS. The time-dependent factors appear to be the most important determinant of post-injury alterations of gene expression in liver regeneration. Gene expression profile after ALPPS showed more similar expression pattern to the PH than the PVL at the early phase of the regeneration. Early transcriptomic changes and predicted upstream regulators that were found in all three procedures included cell cycle associated genes (*E2F1, CCND1, FOXM1, TP53*, and *RB1*), transcription factors (*Myc, E2F1, TBX2, FOXM1*), DNA replication regulators (*CDKN1A, EZH2, RRM2*), G1/S-transition regulators (*CCNB1, CCND1, RABL6*), cytokines and growth factors (*CSF2, IL-6, TNF, HGF, VEGF*, and *EGF*), ATM and p53 signaling pathways. The functional pathway, upstream, and network analyses revealed both unique and overlapping molecular mechanisms and pathways for each surgical procedure. Identification of molecular signatures and regenerative signaling pathways for each surgical procedure further our understanding of key regulators of liver regeneration as well as patient populations that are likely to benefit from each procedure.

## Introduction

While adult hepatocytes are normally quiescent, they show phenomenal replicative potential when parenchymal loss occurs. This has made the liver an excellent model for studying organ regeneration^1–4^. The regenerative process is typically divided into the priming, proliferating and termination phases, occurring approximately at first, third and seventh days after resection, respectively^5^. Clinically, the regenerative ability of livers provides the cornerstone of many surgical treatments for liver diseases; resection of diseased portions seems to improve survival^6^. However, extensive removal of liver parenchyma, which may be necessary in some cases, may overcome the functional reserve of the remaining hepatocyte population, leading to decompensation, failure and even mortality^7^. To avoid this complication, pre-operative iatrogenic induction of hepatocyte proliferation can be attempted to increase the future remnant tissue’s resilience against the anticipated surgical volume loss^7^.

Several options are available to induce the regenerative capacity of the liver. While cellular proliferation may be chemically induced^8^, surgical induction procedures provide additional advantages such as intraoperative assessment of the extent of disease, anatomically-targeted induction, and radical disease resection^7^. These procedures include: Partial hepatectomy (PH), intraoperative portal vein ligation (PVL), percutaneous portal vein embolization (PVE), and associated liver partition and portal vein ligation for staged hepatectomy (ALPPS). Rates at which the remaining tissue regenerates differ between procedures. For example, three to twelve weeks are needed after PVL to achieve adequate regenerative volume compared to 7–8 days in ALPPS^9^. Similarly, Schlegel *et al*. showed a significantly accelerated increase in future liver remnant volume after ALPPS than PVL (1 vs. 4 days, respectively)^10^.

Contrary to other organs where dedicated stem cells replace injured tissues^11^, functional hepatocytes appear to be responsible for liver regeneration^12^. Therefore, major shifts in gene activation and transcription patterns are needed for the transformation from fully-differentiated to highly-proliferative cells. Interestingly, while eventual replacement of lost tissue is a common outcome, contemporary evidence suggests that genomic and transcriptomic alterations may vary based on regeneration-driving factors^8^. Indeed, systematic analysis of genome-wide shifts can identify either common or specific signatures of different procedures, which may provide insights into the key regulators of such a complex process^13^. Additionally, the signatures may prove clinically useful in gauging patients’ treatment responses, planning further treatments, and providing prognostic information.

To date, several reports have investigated the effect of surgical procedures on gene expression patterns in regenerating rat livers after PH^14^, PVL^15^, and ALPPS when compared with controls^10^, as well as PVL vs. ALPPS^10,16,17^. However, to our knowledge, no study has explored the global gene expression patterns in regenerating livers after different regeneration-promoting surgical procedures and performed inter-procedural comparison. Using the power of next-generation RNA sequencing (RNAseq), we provided an overarching comparative analysis of differentially-regulated genes among three surgical groups (PH, PVL, ALPPS versus Sham) in regenerating rat livers, at early regenerating phase (i.e. 24 hours) and late-stage (i.e. 96 hours) post-operatively. Furthermore, we identified both unique and shared molecular mechanisms and signaling pathways for liver regeneration in the investigated surgical procedures.

## Materials and Methods

### Animals

Six to 8 weeks old Male Sprague–Dawley rats weighing 200–250 g were obtained from the Charles-River Laboratories UK Ltd. The animals were housed in an alternating light and dark room with controlled temperature and relative humidity (23 ± 2 °C, 50 ± 10%). They were given standard laboratory rodent chow and free water access, as detailed previously^9^. All the experiments were approved by the local ethics committee (University College London) and conducted according to Home Office guidelines under the UK Animals and Scientific Procedures Act 1986.

### Experimental design and animal groups

Rats were divided into groups and anesthetized under isoflurane administration. A middle line incision was performed on the abdomen. (1) Sham group: Abdomen was closed after manipulation of the liver hilum; (2) PVL group: Portal vein ligation was performed on all branches except the branch to the right median lobe using a size 7–0 nylon thread; and (3) ALPPS group: selective portal vein ligation and liver parenchymal partitioning were performed (4) PH group: A 5–0 nylon thread was tied tightly around the median and left lateral lobes (~70% of the total liver mass) and both lobes were then resected, as described previously^9^. There were three rats for each group at each time point (24 h and 96 h). The detail of surgical operations and experimental grouping is provided in our previous study^9^.

### RNA sequencing analysis

TRIzol (Invitrogen) was used for isolation of total RNA (15 µg) that was utilized for purifying the poly(A)-containing mRNA molecules, RNA amplification, and synthesis of double-stranded cDNAs according to Illumina’s TruSeq RNA Sample Prep guidelines (San Diego, CA). Multiplexed samples were sequenced at 43 bp length on Illumina-based Technology. Triplicate biological replicates were performed for each group. The paired-end reads of each sample were aligned to the rat genome (rn5) using the TopHat^18^. Transcript abundance was estimated by Cufflinks^19^. The quantification and normalization (DESeq method^20^) and further downstream analyses of identification of differentially expressed genes (DEGs) were done by using Strand NGS 2.7 (Strand Life Sciences, India) and PARTEK Genomics Suite (Partek Inc., St. Louis, MO, USA). Significantly regulated genes across different operation types (ALPPS, PVL, PH, and Sham) and two time points (24 h and 96 h) were determined using two-factor analysis of variance (ANOVA) by taking operation type, time and their interactions into the statistical model. Genes exhibiting false discovery rate (FDR)-adjusted *P* value<0.05 and the absolute fold changes (FC) > 2 were considered significant.

### Functional pathway, upstream regulator, and network analyses

Functional, pathway, and gene ontology (GO) enrichment analysis were performed using Database for Annotation, Visualization and Integrated Discovery (DAVID)^21^, Protein Analysis Through Evolutionary Relationships (PANTHER™) classification systems and Ingenuity Pathways Analysis (IPA) (QIAGEN Inc., https://www.qiagenbioinformatics.com/products/ingenuity-pathway-analysis). We also performed upstream regulator, canonical pathways, and gene network analyses using IPA and Network Analyst^22^. The DEGs lists for each surgical procedure for different time points were mapped to its corresponding gene object in the Ingenuity pathway knowledge base and protein-protein interaction networks. A right-tailed Fisher’s exact test was used to calculate a p-value determining the probability that the biological function (or pathway) assigned to that data set is explained by chance alone. The IPA upstream regulator analysis predicts the upstream transcriptional regulators based on the Ingenuity® Knowledge Base by examining how many known targets of the upstream regulators are present in the differentially expressed gene list. An overlap p‐value, based on significant overlap between genes in the list and known targets regulated by the transcriptional regulator, and an activation z‐score are computed. The predicted activation state and activation z-score are based on the direction of fold change values that we observed in the gene expression data. The activation z‐score is to infer likely activation states, “activated” or “inhibited”, of upstream transcriptional regulators. It was considered significantly activated (or inhibited) with an overlap p-value ≤ 0.05 and an z-score ≥ 2.0 (or ≤ −2.0).

## Results

### Global gene expression changes in regenerating livers after ALPPS, PVL and PH

**Figure 1:**
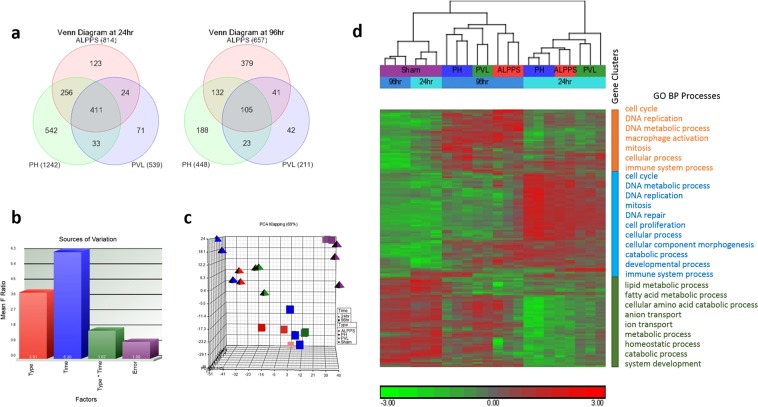
(**a**) Venn diagrams representing the differentially expressed genes specific or common among ALPPS, PH and PVL at 24 h and 96 h, respectively. **(b)** Sources of variation in the data matrix. The x-axis shows the components of the 2-way ANOVA model and the y-axis shows the mean signal to noise ratio. **(c)** Unsupervised PCA analysis. Different colors indicate different surgical types and shapes indicate different time points. **(d)** Two-dimensional hierarchical clustering of genes, that are significant in at least one surgical procedure (with respect to sham group) at any of the two time points, and samples. The figure shows the most associated GO biological processes for each cluster of genes. Red and green denote highly and weakly expressed genes, respectively.

Global transcriptome changes associated with the surgical procedures at each time point were measured by RNAseq approach. The analysis generated 14,989,372 to 120,655,689 reads, which represented 88.5% of the genome (rn5), and 31,399 transcripts (Supplementary Table S1). There were 2,014 genes that showed a significant change in at least one surgical procedure (with respect to sham group) at either of the two time points (Fig. 1a). The variation in the data matrix was mostly due to the time effect, followed by the operation type (Fig. 1b). Principal components analyses (PCA) and unsupervised hierarchical clustering separated the samples according to two time points (24 h and 96 h) and surgical procedure types (Fig. 1c,d, respectively). Genes involved in cell cycle, mitosis, developmental process, and DNA replication were up-regulated in three surgical groups at 24 h with highest increase was observed in PH and ALPPS compared to the PVL group, which were later repressed at 96 h (Fig. 1d). Macrophage activation, cell adhesion, immune process, ion transport, and lipid metabolic process related genes had low level of expression at 24 h, but were up-regulated at 96 h post-operation (Fig. 1d).

### Inter-procedural similarities/differences at 24 h and 96 h post operations

Since the time had the greatest effect on the expression, we next compared the transcriptomes of the procedures at each time point separately. Differentially expressed genes (DEGs) that are specific or commonly dysregulated between the procedures at 24 h and 96 h were obtained using the Venn diagram approach (Fig. 1a). The PCA and hierarchical clustering analyses of the significant genes (FDR < 5% and FC > 2) separated the samples according to the surgical types at each time points (Supplementary Fig. S1).

At 24 h, the PH vs Sham produced the largest number of DEGs, followed by ALPPS vs Sham (Fig. 1a, Supplementary Table S2). There were 1242 DEGs for the PH (703↑ up-regulated, 539↓ down-regulated), 814 for the ALPPS group (536↑, 278↓), and 539 for the PVL (428↑, 111↓), and 411 DEGs (351↑, 60↓) were common to all surgical procedures. The arrow represents an increase (↑) or decrease (↓) in fold compared to Sham. Comparing different procedures, 123, 542, and 71 genes were specifically expressed in ALPPS, PH, and PVL, respectively (Fig. 1a). There were more DEGs commonly shared between the ALPPS and the PH than with the PVL, especially at 24 h. The list of DEGs shared and unique to each procedure is given in Supplementary Table S2. The ALPPS showed more similar expression pattern to the PH than the PVL especially at the early phase of the regeneration at 24 h post operation (Supplementary Fig. S1).

**Figure 2:**
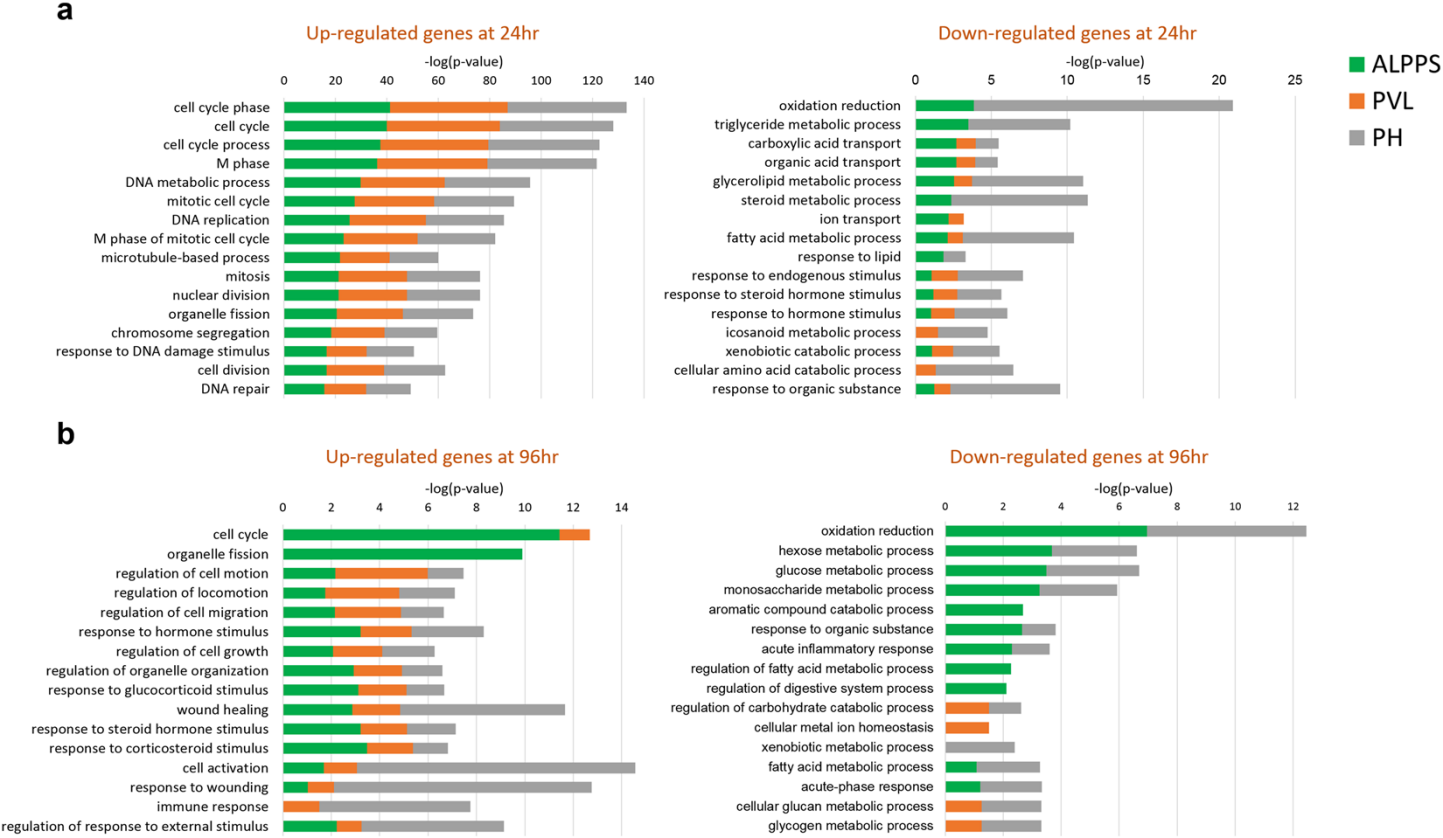
Bar chart of the most significant GO biological processes that are associated with up- and down-regulated genes for each surgical procedure at 24h (**a**) and 96h (**b**). X-axis represents the statistical significance of the enrichment (–log10(p-value)). Color-coding represents different surgical types.

We further performed an extensive comparison of functional enrichment and gene networks for the three surgical procedures using different bioinformatics tools to further investigate molecular similarities/differences among the procedures. Gene ontology enrichment analysis revealed that genes related to cell cycle, mitotic cell cycle, M phase, and DNA replication and repair were significantly enriched among the up-regulated genes at 24 h for all surgical procedures (Supplementary Fig. S1). However, genes related to oxidation-reduction, triglyceride, and steroid metabolic processes were the most critically enriched among the down-regulated genes in ALPPS and PH only (Fig. 2a, Table 1, and Supplementary Fig. S4).

**Table 1:** GO Biological Processes that are enriched in DEGs in ALPPS, PVL and PH.

GO Biological Process Term	24 hours						96 hours					
	ALPPS		PVL		PH		ALPPS		PVL		PH	
	FE*	p-value	FE*	p-value	FE*	p-value	FE*	p-value	FE*	p-value	FE*	p-value
DNA metabolic process	4.7	7.0E-21	5.8	8.8E-24	3.3	3.8E-17	1.6	3.3E-02	—	—	—	—
cell cycle	3.0	6.3E-19	3.8	1.7E-23	2.5	1.3E-18	1.9	2.4E-05	—	—	—	—
DNA replication	6.9	2.0E-18	9.2	1.7E-22	4.5	1.4E-14	2.6	2.0E-03	—	—	—	—
chromosome segregation	7.3	1.5E-13	8.9	2.5E-14	5.4	1.6E-13	3.4	5.8E-04	—	—	2.7	2.4E-02
DNA repair	5.0	2.1E-11	6.2	1.1E-12	3.3	1.8E-08	–	–	—	—	—	—
mitosis	3.4	1.3E-10	4.4	4.6E-13	2.8	5.6E-11	2.1	9.3E-04	—	—	—	—
catabolic process	2.2	2.1E-07	2.3	1.4E-06	1.7	1.5E-05	1.8	2.5E-04	—	—	—	—
cellular process	1.2	9.6E-07	1.2	4.1E-06	1.1	6.6E-03	1.2	1.7E-04	—	—	1.3	2.1E-07
ion transport	2.2	3.5E-04	—	—	1.6	5.0E-03	2.4	5.0E-05	—	—	—	—
cytokinesis	2.9	2.7E-03	3.2	4.5E-03	2.0	1.9E-02	2.4	2.2E-02	—	—	—	—
metabolic process	1.2	6.1E-06	1.3	1.7E-06	1.2	6.3E-07	–	–	—	—	—	—
cellular component movement	2.6	3.4E-06	2.5	6.1E-05	1.9	8.0E-05	2.1	6.3E-04	—	—	2.7	2.9E-05
lipid metabolic process	1.6	1.6E-02	—	—	2.6	2.8E-13	2.1	3.2E-05	—	—	2.3	1.1E-04
response to stress	1.6	2.2E-03	1.7	1.1E-03	1.4	6.1E-03	—	—	—	—	1.5	1.9E-02
anion transport	2.3	1.7E-03	—	—	1.7	1.4E-02	2.9	2.6E-05	—	—	—	—
cellular component morphogenesis	2.0	1.3E-04	1.7	1.1E-02	—	—	2.3	8.7E-06	3.1	8.7E-05	2.3	2.6E-04
macrophage activation	—	—	—	—	—	—	3.7	8.1E-05	3.5	3.0E-02	3.4	3.0E-03
system development	—	—	—	—	—	—	1.6	9.5E-04	1.8	1.5E-02	1.7	2.4E-03
immune system process	—	—	—	—	—	—	1.4	1.2E-02	—	—	2.2	2.3E-08
developmental process	—	—	—	—	—	—	1.3	1.5E-02	—	—	1.7	6.1E-05
cell proliferation	—	—	—	—	—	—	2.2	2.6E-02	—	—	6.1	6.1E-09
signal transduction	—	—	—	—	—	—	–	–	—	—	1.7	5.5E-07
cell communication	—	—	—	—	—	—	–	–	—	—	1.6	1.7E-06
cell adhesion	—	—	—	—	—	—	2	6.5E-04	—	—	2.6	1.2E-05

Abbreviations: DEG, differentially expressed gene; FE, Fold Enrichment is the number of DEGs involved in each GO biological process term divided by the expected number. – Denotes non-significant term.

At 96 h post-operatively, there were 657 (493↑, 164↓), 448 (387↑, 61↓), and 211 (179↑, 32↓) genes for ALPPS, PH and PVL, respectively, when compared with the Sham livers (FDR < 5% and FC > 2) (Fig. 1a, Supplementary Table S3). The ALPPS vs Sham produced the largest number of DEGs. Genes responsible for cell cycle, ion transport, mitosis, and organelle fission seemed to be highly enriched in the up-regulated genes in the ALPPS; genes responsible for cell activation, response to wounding, and immune response seemed to be the most significantly enriched in the PH. The down-regulated genes at 96 h, especially for the ALPPS and PH, were enriched for oxidation-reduction, metabolic process and inflammatory response (Fig. 2b). The cell adhesion, immune system process, cellular process and cell proliferation associated genes were significantly enriched after ALPPS and PH, and macrophage activation, system development, and cellular component and morphogenesis were significant after all the procedures at 96 h (Table 1).

### Common gene signatures of liver regeneration at 24 h and 96 h

**Figure 3:**
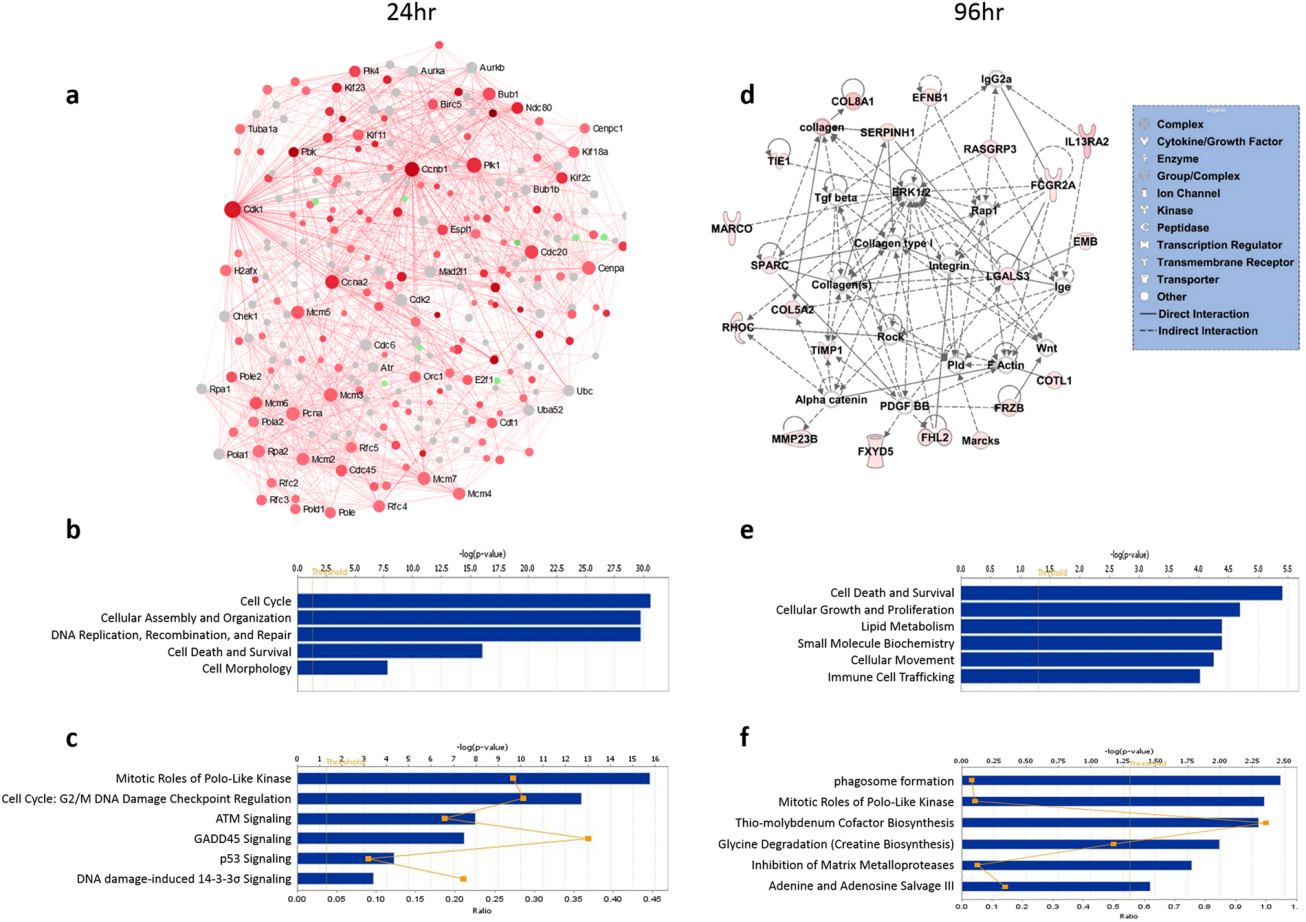
Gene interaction network, functional, and canonical pathway analyses (**a–c**, respectively) at 24h and (**d–f**, respectively) at 96h of common DEGs among ALPPS, PH and PVL. Red and green denote up- and down-regulated genes, respectively, and grey indicates direct interactors of the DEGs. The sizes of nodes (in **a**) are proportional to their betweenness centrality values^22^. Straight and dashed lines (in **d**) represent direct or indirect gene to gene interactions, respectively. The color intensity is correlated with fold change. X-axis (in bar graphs) indicates the significance (–log10(p-value)) of the functional/pathway association. The threshold line represents a P value of 0.05.The network (in **d**) and functional/pathway analyses were generated using IPA (QIAGEN Inc., https://www.qiagenbioinformatics.com/products/ingenuity-pathway-analysis).

Among the all surgical procedures, 411 DEGs (351↑, 60↓) and 105 DEGs (99↑, 6↓) were dysregulated at 24 h and 96 h, respectively (FDR < 5% and absolute FC > 2) (Supplementary Fig. S2A, Tables S2 and S3, respectively). GO enrichment and functional analyses of DEGs at 24 h indicated involvement of genes related to cell cycle, DNA replication and repair, cell division, mitosis, and DNA metabolic process (Table 1, Fig. 3, and Supplementary Fig. S2B). Furthermore, the gene network, functional and pathway analyses revealed potentially important roles of *Ccnb1, Cdk1, E2f1, Ccna2, Rrm2, Lcn2*, NF-κB, and *Vegf* in liver regeneration and alterations in cell cycle and p53 pathways (Fig. 3a–c and Supplementary Fig. S3).

At 96 h, the DEGs that are commonly regulated in all surgical groups were mostly up-regulated (94%) and mainly involved in cellular movement, system development, extracellular matrix organization, and immune response (Supplementary Fig. S2B). The gene ontology and network analyses indicated enrichment of genes related to cell death and survival, cellular growth and proliferation, lipid metabolism, and immune cell trafficking, including genes such as *LGALS3, FCGR2A, SPARC*, *Integrin*, and *Collagens* (p-value <0.01) (Fig. 3d–f), at 96 h post-operations.

### Functional, pathway and gene network comparison of temporal changes after ALPPS, PH and PVL

We further performed an extensive comparison of functional enrichment and gene networks for the three surgical procedures using different bioinformatics tools to further investigate molecular similarities/differences of the procedures as well as to investigate temporal changes within each surgical procedure at two time points.

Genes related to cell cycle, mitotic cell cycle, M phase, and DNA replication and repair were significantly enriched among the up-regulated genes at 24 h for all surgical procedures (Fig. 2a, Table 2, and Supplementary Fig. S4). However, genes related to oxidation-reduction, triglyceride, and steroid metabolic processes were the most critically enriched among the down-regulated genes in ALPPS and PH only. On the other hand, at 96 h, genes responsible for cell cycle and organelle fission seemed to be highly enriched in the up-regulated genes in the ALPPS; genes responsible for cell activation, response to wounding, and immune response seemed to be the most significantly enriched in the PH. The down-regulated genes at 96 h, especially for the ALPPS and PH, were enriched for oxidation-reduction, metabolic process and inflammatory response (Fig. 2b).

**Table 2:** Significantly altered canonical pathways, molecular and cellular functions, and predicted upstream regulators associated with DEGs in ALPPS, PVL and PH at 24 h and 96 h.

ALPPS at 24 hr			ALPPS at 96 hr		
Canonical Pathways	p-value	Overlap	Canonical Pathways	p-value	Overlap
Cell Cycle Control of Chromosomal Replication	1.51E-14	51.9% 14/27	LPS/IL-1 Mediated Inhibition of RXR Function	8.00E-06	9.5% 21/221
Mitotic Roles of Polo-Like Kinase	1.07E-14	30.3% 20/66	Mitotic Roles of Polo-Like Kinase	6.53E-06	16.7% 11/66
Role of BRCA1 in DNA Damage Response	3.84E-12	24.4% 19/78	FXR/RXR Activation	1.10E-05	11.9% 15/126
Cell Cycle: G2/M DNA Damage Checkpoint Regulation	1.37E-12	32.7% 16/49	Hepatic Fibrosis / Hepatic Stellate Cell Activation	2.47E-04	8.7% 16/183
**Upstream Regulators**	**p-value**	**Predicted Activation**	**Upstream Regulators**	**p-value**	**Predicted Activation**
E2F1	4.93E-41	Activated	ERBB2	4.36E-22	Activated
CCND1	2.35E-40	Activated	TGFB1	5.96E-22	Activated
RABL6	6.32E-38	Activated	VEGF	8.37E-21	Activated
CDKN1A	2.73E-57	Inhibited	CSF2	7.02E-16	Activated
TP53	6.98E-53	Inhibited	CDKN1A	3.22E-24	Inhibited
**Molecular and Cellular Functions**	**p-value range**	**# Molecules**	**Molecular and Cellular Functions**	**p-value range**	**# Molecules**
Cell Cycle	2.22E-05 - 5.88E-29	209	Cell Death and Survival	4.43E-04 - 4.11E-16	257
Cellular Assembly and Organization	2.88E-05 - 6.87E-26	186	Cell Cycle	4.19E-04 - 4.78E-15	135
DNA Replication, Recombination, and Repair	2.22E-05 - 6.87E-26	173	Cellular Assembly and Organization	4.64E-04 - 4.78E-15	176
Cell Death and Survival	2.88E-05 - 1.12E-18	278	DNA Replication, Recombination, and Repair	4.19E-04 - 4.78E-15	107
Cell Morphology	2.88E-05 - 4.40E-15	145	Cellular Growth and Proliferation	4.61E-04 - 3.22E-15	287
**Physiological System Development and Function**	**p-value range**	**# Molecules**	**Physiological System Development and Function**	**p-value range**	**# Molecules**
Organismal Development	1.15E-06 - 4.56E-09	19	Cardiovascular System Development and Function	2.79E-04 - 5.36E-09	119
Connective Tissue Development and Function	1.74E-05 - 1.02E-10	92	Tissue Development	3.41E-04 - 6.93E-09	211
Tissue Development	2.30E-05 - 5.37E-10	106	Immune Cell Trafficking	2.77E-04 - 1.04E-08	85
Tissue Morphology	2.22E-05 - 4.56E-09	30	Hematological System Development and Function	2.77E-04 - 3.13E-08	112
Embryonic Development	1.15E-06 - 4.56E-09	19	Organismal Development	3.38E-04 - 9.93E-08	128
**Tox Lists**	**p-value**	**Overlap**	**Tox Lists**	**p-value**	**Overlap**
Cell Cycle: G2/M DNA Damage Checkpoint Regulation	3.86E-12	30.8% 16/52	Renal Necrosis/Cell Death	2.36E-07	7.7% 40/519
Liver Proliferation	4.84E-06	9.6% 22/228	LPS/IL-1 Mediated Inhibition of RXR Function	1.49E-07	10.3% 26/253
Aryl Hydrocarbon Receptor Signaling	9.04E-07	11.9% 19/159	FXR/RXR Activation	1.10E-05	11.9% 15/126
CAR/RXR Activation	2.58E-06	27.6% 8/29	Cardiac Hypertrophy	1.39E-06	7.8% 34/435
**PVL at 24 hr**			**PVL at 96 hr**		
**Canonical Pathways**	**p-value**	**Overlap**	**Canonical Pathways**	**p-value**	**Overlap**
Cell Cycle Control of Chromosomal Replication	3.16E-16	51.9% 14/27	GADD45 Signaling	9.30E-04	15.8% 3/19
Mitotic Roles of Polo-Like Kinase	9.19E-16	28.8% 19/66	Purine Ribonucleosides Degradation to Ribose-1-phosphate	2.85E-03	25.0% 2/8
Role of BRCA1 in DNA Damage Response	3.60E-13	23.1% 18/78	GDP-glucose Biosynthesis	3.64E-03	22.2% 2/9
Estrogen-mediated S-phase Entry	2.95E-12	45.8% 11/24	Glucose and Glucose-1-phosphate Degradation	4.52E-03	20.0% 2/10
Cell Cycle: G2/M DNA Damage Checkpoint Regulation	3.67E-13	30.6% 15/49	Mitotic Roles of Polo-Like Kinase	4.83E-03	6.1% 4/66
**Upstream Regulators**	**p-value**	**Predicted Activation**	**Upstream Regulators**	**p-value**	**Predicted Activation**
E2F1	6.82E-48	Activated	TGFB1	7.66E-11	Activated
RABL6	1.67E-42	Activated	CSF2	1.20E-07	Activated
CCND1	2.68E-41	Activated	IL4	3.51E-06	Activated
CDKN1A	9.47E-67	Inhibited	SMARCA4	5.42E-06	Activated
TP53	1.14E-63	Inhibited	MYCN	8.83E-04	Inhibited
**Molecular and Cellular Functions**	**p-value range**	**# Molecules**	**Molecular and Cellular Functions**	**p-value range**	**# Molecules**
Cell Cycle	1.57E-05 - 4.47E-38	202	Cell Death and Survival	9.04E-03 - 1.69E-09	96
Cellular Assembly and Organization	1.48E-05 - 3.39E-34	163	Cellular Growth and Proliferation	8.25E-03 - 3.76E-09	106
DNA Replication, Recombination, and Repair	1.43E-05 - 3.39E-34	160	Cellular Assembly and Organization	8.66E-03 - 3.93E-07	62
Cell Death and Survival	1.47E-05 - 5.70E-21	224	Cell Morphology	9.04E-03 - 7.52E-07	67
Cell Morphology	1.44E-05 - 5.49E-17	107	Cell-To-Cell Signaling and Interaction	8.89E-03 - 1.32E-06	44
**Physiological System Development and Function**	**p-value range**	**# Molecules**	**Physiological System Development and Function**	**p-value range**	**# Molecules**
Reproductive System Development and Function	1.43E-05 - 9.48E-12	52	Tissue Development	8.89E-03 - 3.93E-07	92
Organismal Development	6.34E-09 - 4.28E-11	19	Lymphoid Tissue Structure and Development	8.18E-03 - 4.34E-06	35
Connective Tissue Development and Function	1.07E-05 - 8.94E-11	81	Organ Morphology	8.89E-03 - 4.34E-06	38
Tissue Morphology	5.72E-06 - 4.28E-11	50	Cardiovascular System Development and Function	7.67E-03 - 1.32E-06	47
Embryonic Development	1.14E-05 - 4.28E-11	34	Tissue Morphology	8.46E-03 - 4.34E-06	65
**Tox Lists**	**p-value**	**Overlap**	**Tox Lists**	**p-value**	**Overlap**
Cell Cycle: G2/M DNA Damage Checkpoint Regulation	9.76E-13	28.8% 15/52	Nongenotoxic Hepatocarcinogenicity Biomarker Panel	6.98E-05	18.2% 4/22
Increases Liver Hyperplasia/Hyperproliferation	1.29E-06	12.6% 13/103	Recovery from Ischemic Acute Renal Failure (Rat)	3.63E-04	21.4% 3/14
Aryl Hydrocarbon Receptor Signaling	6.67E-08	11.3% 18/159	Cardiac Hypertrophy	6.20E-04	3.0% 13/435
Cell Cycle: G1/S Checkpoint Regulation	5.04E-07	16.7% 11/66	Increases Renal Damage	1.52E-03	6.2% 5/81
**PH at 24 hr**			**PH at 96 hr**		
**Canonical Pathways**	**p-value**	**Overlap**	**Canonical Pathways**	**p-value**	**Overlap**
Cell Cycle Control of Chromosomal Replication	2.21E-15	63.0% 17/27	Production of Nitric Oxide and Reactive Oxygen Species in Macrophages	5.40E-09	10.4% 20/193
Role of BRCA1 in DNA Damage Response	2.90E-12	30.8% 24/78	LXR/RXR Activation	5.62E-09	13.2% 16/121
Mitotic Roles of Polo-Like Kinase	3.83E-12	33.3% 22/66	Acute Phase Response Signaling	1.18E-07	10.1% 17/169
Hereditary Breast Cancer Signaling	4.47E-11	21.8% 31/142	Phagosome Formation	1.08E-05	9.8% 12/122
Cell Cycle: G2/M DNA Damage Checkpoint Regulation	5.42E-11	36.7% 18/49	Fc Receptor-mediated Phagocytosis in Macrophages and Monocytes	2.70E-05	10.8% 10/93
**Upstream Regulators**	**p-value**	**Predicted Activation**	**Upstream Regulators**	**p-value**	**Predicted Activation**
CCND1	4.01E-37	Activated	TNF	4.33E-30	Activated
E2F1	5.13E-34	Activated	IFNG	2.25E-22	Activated
RABL6	1.42E-33	Activated	TP53	1.60E-21	Activated
CDKN1A	2.64E-47	Inhibited	IL6	6.78E-20	Activated
TP53	7.48E-47	Inhibited	IL1B	4.93E-19	Activated
**Molecular and Cellular Functions**	**p-value range**	**# Molecules**	**Molecular and Cellular Functions**	**p-value range**	**# Molecules**
Cell Cycle	6.16E-05 - 1.88E-28	281	Cellular Movement	8.64E-06 - 1.06E-28	161
Cellular Assembly and Organization	6.85E-05 - 1.88E-28	163	Cellular Growth and Proliferation	1.01E-05 - 2.44E-24	228
DNA Replication, Recombination, and Repair	6.85E-05 - 1.88E-28	243	Cell Death and Survival	9.36E-06 - 3.12E-23	201
Cell Death and Survival	7.54E-05 - 6.73E-17	420	Cell-To-Cell Signaling and Interaction	7.47E-06 - 4.76E-20	133
Cell Morphology	1.58E-05 - 4.68E-15	229	Cellular Function and Maintenance	6.05E-06 - 4.83E-17	187
**Physiological System Development and Function**	**p-value range**	**# Molecules**	**Physiological System Development and Function**	**p-value range**	**# Molecules**
Organismal Survival	1.95E-06 - 3.67E-09	292	Hematological System Development and Function	9.36E-06 - 1.66E-27	177
Reproductive System Development and Function	3.04E-05 - 3.16E-10	27	Immune Cell Trafficking	8.64E-06 - 4.34E-28	120
Connective Tissue Development and Function	5.16E-05 - 8.45E-11	132	Cardiovascular System Development and Function	9.16E-06 - 2.79E-19	120
Tissue Development	6.77E-05 - 8.45E-11	117	Organismal Development	7.85E-06 - 5.64E-17	154
Embryonic Development	7.74E-06 - 3.42E-07	27	Tissue Morphology	9.16E-06 - 8.60E-20	158
**Tox Lists**	**p-value**	**Overlap**	**Tox Lists**	**p-value**	**Overlap**
Cell Cycle: G2/M DNA Damage Checkpoint Regulation	1.71E-10	34.6% 18/52	Positive Acute Phase Response Proteins	3.28E-10	33.3% 10/30
LPS/IL-1 Mediated Inhibition of RXR Function	2.44E-09	15.8% 40/253	LXR/RXR Activation	7.16E-09	13.0% 16/123
Fatty Acid Metabolism	5.33E-09	21.4% 25/117	Cardiac Necrosis/Cell Death	1.60E-06	7.3% 20/273
Aryl Hydrocarbon Receptor Signaling	1.51E-08	18.2% 29/159	Cardiac Hypertrophy	6.76E-07	6.2% 27/435
Xenobiotic Metabolism Signaling	2.97E-06	11.9% 42/352	Acute Renal Failure Panel (Rat)	5.80E-06	14.5% 9/62

The DEGs for each procedure at each time point were mapped to gene interaction networks in order to obtain deeper insights into the interactions of these genes among various pathways. The functional, pathway and gene network analyses highlighted the potentially critical genes, biological processes, and signaling pathways for the temporal changes within ALLPS, PVL, and PH groups (Table 2, Supplementary Fig. S4). Cell cycle, mitosis, and DNA replication, process-related genes, such as *Cdk1, E2f1, Ezh2, Ccnb1, Cdkn1a*, *Myc*, and p53 pathways were significantly regulated at 24 h in all procedures. The networks for ALLPS and PH were more similar than PVL at early phase of the regeneration, corroborating with the clustering results in Fig. 1 and Supplementary Fig. S1. At 96 h, cell proliferation, cell cycle, mitosis and cell division remained active in the ALPPS when compared to the PH and PVL. Genes related to macrophage activation, anion transport, immune response, lipid metabolic process were also significantly regulated in the ALPPS (Table 1, Supplementary Fig. S4). Significantly altered pathways included the FXR/RXR activation, cell cycle and integrin signaling pathways in the ALPPS group at 96 h. On the other hand, cellular movement, immune cell trafficking, and signal transduction related genes and T-cell activation and Ras pathways were significantly altered in the PH group at 96 h. Top five significant molecular and cellular functions, canonical pathways, and predicted upstream regulators (activated/inhibited) for each surgical procedure at two time points are given in Table 2. Furthermore, the complete list of significant PANTHER and canonical pathways are given in Table 3 and Supplementary Table S4, respectively.

**Table 3:** Significantly altered pathways in ALPPS, PVL and PH at 24 h and 96 h post operation.

ALPPS at 24 hr				ALPPS at 96 hr			
PANTHER Pathways	DEGs	FE	P-value	PANTHER Pathways	DEGs	FE	P-value
Proline biosynthesis	2	16.6	6.7E-03	N-acetylglucosamine metabolism	3	16.8	8.3E-04
De novo pyrimidine deoxyribonucleotide biosynthesis	6	15.3	3.5E-06	Adenine and hypoxanthine salvage pathway	3	12.6	1.9E-03
Formyltetrahydroformate biosynthesis	3	14.2	1.3E-03	Pyruvate metabolism	5	9.9	1.8E-04
DNA replication	11	7.9	2.4E-07	Formyltetrahydroformate biosynthesis	2	9.6	1.9E-02
Plasminogen activating cascade	3	5.9	1.5E-02	5-Hydroxytryptamine degredation	3	4.8	2.6E-02
Cell cycle	4	5.3	7.3E-03	Axon guidance mediated by semaphorins	3	4.2	3.6E-02
Cytoskeletal regulation by Rho GTPase	10	4.0	2.7E-04	Heterotrimeric G-protein signaling pathway-Gq alpha and Go alpha mediated pathway	13	3.6	9.5E-05
Blood coagulation	5	3.5	1.5E-02	Angiotensin II-stimulated signaling through G proteins and beta-arrestin	4	3.2	3.8E-02
p53 pathway	12	3.2	5.6E-04	Heterotrimeric G-protein signaling pathway-Gi alpha and Gs alpha mediated pathway	12	2.5	4.2E-03
p53 pathway feedback loops 2	5	2.8	3.7E-02	Alzheimer disease-presenilin pathway	9	2.3	2.1E-02
Alzheimer disease-presenilin pathway	11	2.7	2.9E-03	Integrin signalling pathway	13	2.2	7.5E-03
**PVL at 24 hr**				**PVL at 96 hr**			
**PANTHER Pathways**	**DEGs**	**FE**	**P-value**	**PANTHER Pathways**	**DEGs**	**FE**	**P-value**
Formyltetrahydroformate biosynthesis	3	19.1	5.7E-04	p53 pathway	4	3.3	3.6E-02
De novo pyrimidine deoxyribonucleotide biosynthesis	5	17.2	1.4E-05	Axon guidance mediated by semaphorins	2	8.6	2.3E-02
Phenylethylamine degradation	2	12.8	1.1E-02	Beta3 adrenergic receptor signaling pathway	2	7.1	3.3E-02
DNA replication	11	10.7	1.3E-08	Opioid prodynorphin pathway	2	6.2	4.2E-02
Cell cycle	4	7.1	2.6E-03	Cortocotropin releasing factor receptor signaling pathway	2	6.2	4.2E-02
p53 pathway feedback loops 2	7	5.2	4.8E-04	Opioid proopiomelanocortin pathway	2	6.1	4.4E-02
De novo purine biosynthesis	3	4.6	2.8E-02	Opioid proenkephalin pathway	2	6.1	4.4E-02
p53 pathway	11	3.9	1.7E-04	5HT4 type receptor mediated signaling pathway	2	6.1	4.4E-02
Blood coagulation	4	3.8	2.2E-02	Heterotrimeric G-protein signaling pathway-Gq alpha and Go alpha mediated pathway	4	3.4	3.1E-02
Cytoskeletal regulation by Rho GTPase	5	2.7	4.1E-02				
**PH at 24 hr**				**PH at 96 hr**			
**PANTHER Pathways**	**DEGs**	**FE**	**P-value**	**PANTHER Pathways**	**DEGs**	**FE**	**P-value**
Proline biosynthesis	3	14.3	1.3E-03	N-acetylglucosamine metabolism	3	25.1	2.6E-04
Phenylethylamine degradation	3	8.1	6.3E-03	Pyruvate metabolism	4	11.8	4.1E-04
Formyltetrahydroformate biosynthesis	3	8.1	6.3E-03	Plasminogen activating cascade	3	8.9	5.0E-03
Vitamin B6 metabolism	2	7.6	2.9E-02	Axon guidance mediated by semaphorins	4	8.4	1.5E-03
Salvage pyrimidine deoxyribonucleotides	2	7.6	2.9E-02	Blood coagulation	5	5.4	2.7E-03
De novo pyrimidine deoxyribonucleotide biosynthesis	5	7.3	7.1E-04	Heterotrimeric G-protein signaling pathway-Gq alpha and Go alpha mediated pathway	11	4.6	4.1E-05
DNA replication	16	6.6	6.5E-09	Enkephalin release	3	4.2	3.6E-02
Pyruvate metabolism	4	4.5	1.3E-02	Ras Pathway	6	3.8	5.8E-03
Androgen/estrogene/progesterone biosynthesis	3	4.1	3.9E-02	VEGF signaling pathway	5	3.3	1.9E-02
Cell cycle	5	3.8	1.1E-02	Heterotrimeric G-protein signaling pathway-Gi alpha and Gs alpha mediated pathway	10	3.1	1.9E-03
Blood coagulation	9	3.6	1.1E-03	Interleukin signaling pathway	6	3.1	1.5E-02
5-Hydroxytryptamine degredation	4	3.6	2.6E-02	Cytoskeletal regulation by Rho GTPase	5	3.0	2.6E-02
Cytoskeletal regulation by Rho GTPase	12	2.8	1.9E-03	Angiogenesis	10	2.8	3.9E-03
p53 pathway feedback loops 2	8	2.5	1.6E-02	PDGF signaling pathway	8	2.7	1.1E-02
p53 pathway	15	2.3	3.5E-03	CCKR signaling map	9	2.6	9.6E-03
				T cell activation	5	2.5	5.0E-02
				Inflammation mediated by chemokine and cytokine signaling pathway	13	2.5	2.4E-03

Abbreviations: DEGs, number of differentially expressed genes with respect to Sham; FE, Fold Enrichment is the number of DEGs involved in each pathway divided by the expected number.

### Temporal pattern of upstream transcriptional regulators after ALPPS, PH and PVL

**Figure 4:**
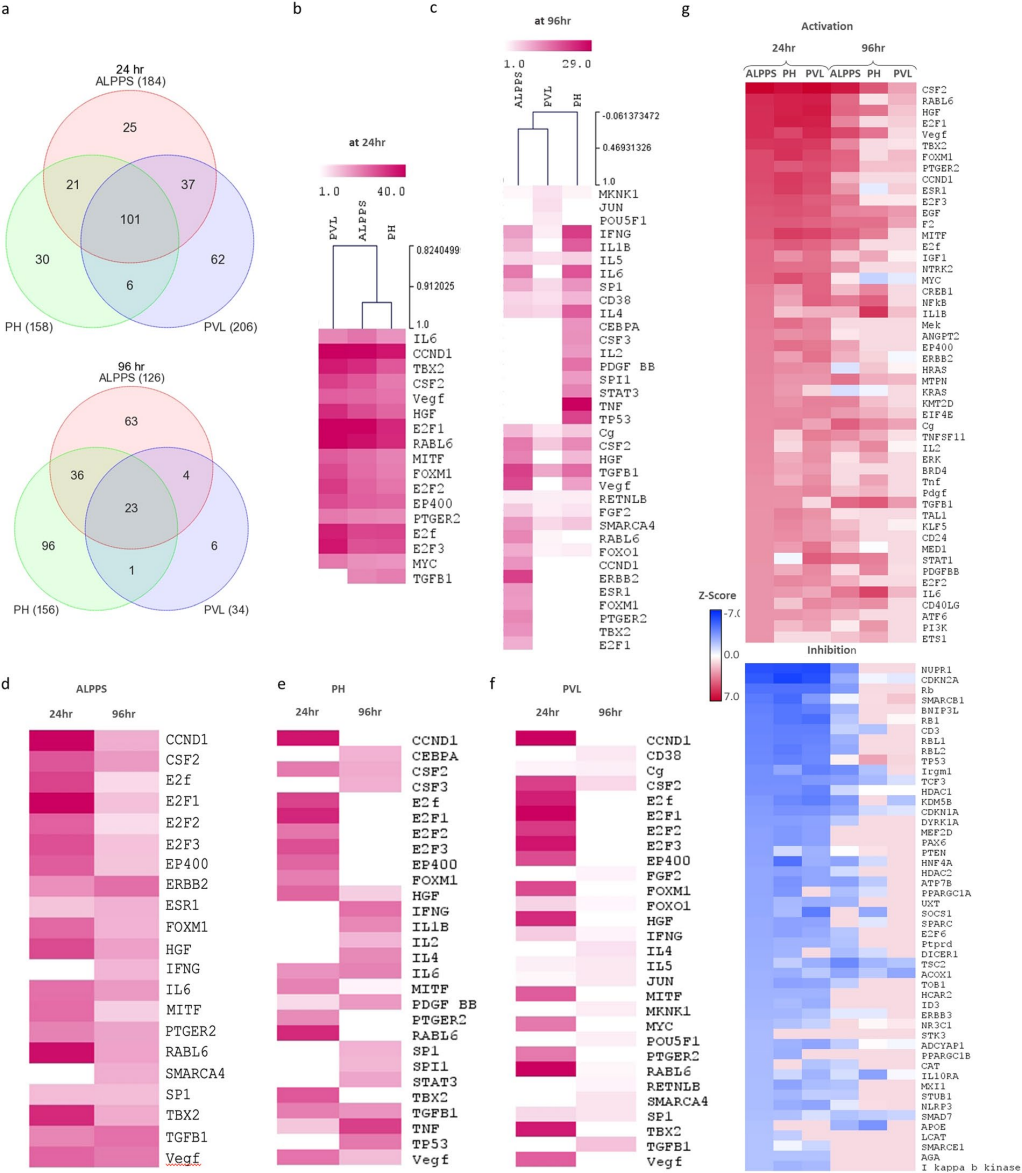
The upstream regulator analyses. (**a**) Venn diagrams representing the overlap of predicted upstream regulators that are unique or common among ALPPS, PH and PVL at 24 h (top) and 96 h (bottom). **(b,c)** Inter-procedural variations at 24 h and 96 h, respectively. (**d–f**) Temporal variations after ALPPS, PH and PVL, respectively. Heatmaps for union of top 15 activated upstream regulators in ALPPS, PVL and PH at 24 h and 96 h are used. Color scale shows significance by –log10(p-value). **(g)** Heatmap that displays surgical procedure as well as temporal changes for the top 50 activated/inhibited upstream regulators. The upstream regulators are ordered according to significance in ALPPS procedure. The color-key is for the z-score. Data were analyzed through the use of IPA (QIAGEN Inc., https://www.qiagenbioinformatics.com/products/ingenuitypathway-analysis).

We identified the transcriptional regulators likely to be involved in the regenerating liver after each surgical procedure at two time points (Fig. 4). The IPA upstream regulator analytic identifies the upstream transcriptional regulators and mechanistic networks that can explain the gene expression changes observed after each procedure. There were 184, 158, and 206 upstream regulators predicted to be activated or inhibited after ALPPS, PH, and PVL, respectively at 24 hr (Fig. 4a). There was a noteworthy overlap of transcriptional regulators between different procedures at 24 h. The inter-procedural variations in ALPPS, PVL and PH are displayed at 24 h and 96 h (in Fig. 4b,c, respectively) for the top 15 activated upstream regulators. The union of top 15 activated upstream regulators in ALPPS, PVL and PH at 24 h and 96 h is used for the heatmap. Similarly, the temporal variations in each procedure are also investigated for ALPPS, PH and PVL, in Fig. 4d–f, respectively. Figure 4 also displays the heatmap of the top 50 activated/inhibited upstream regulators across all samples to investigate the temporal changes for the three procedures (complete list is given in Supplementary Table S5) (Fig. 4g).

**Figure 5:**
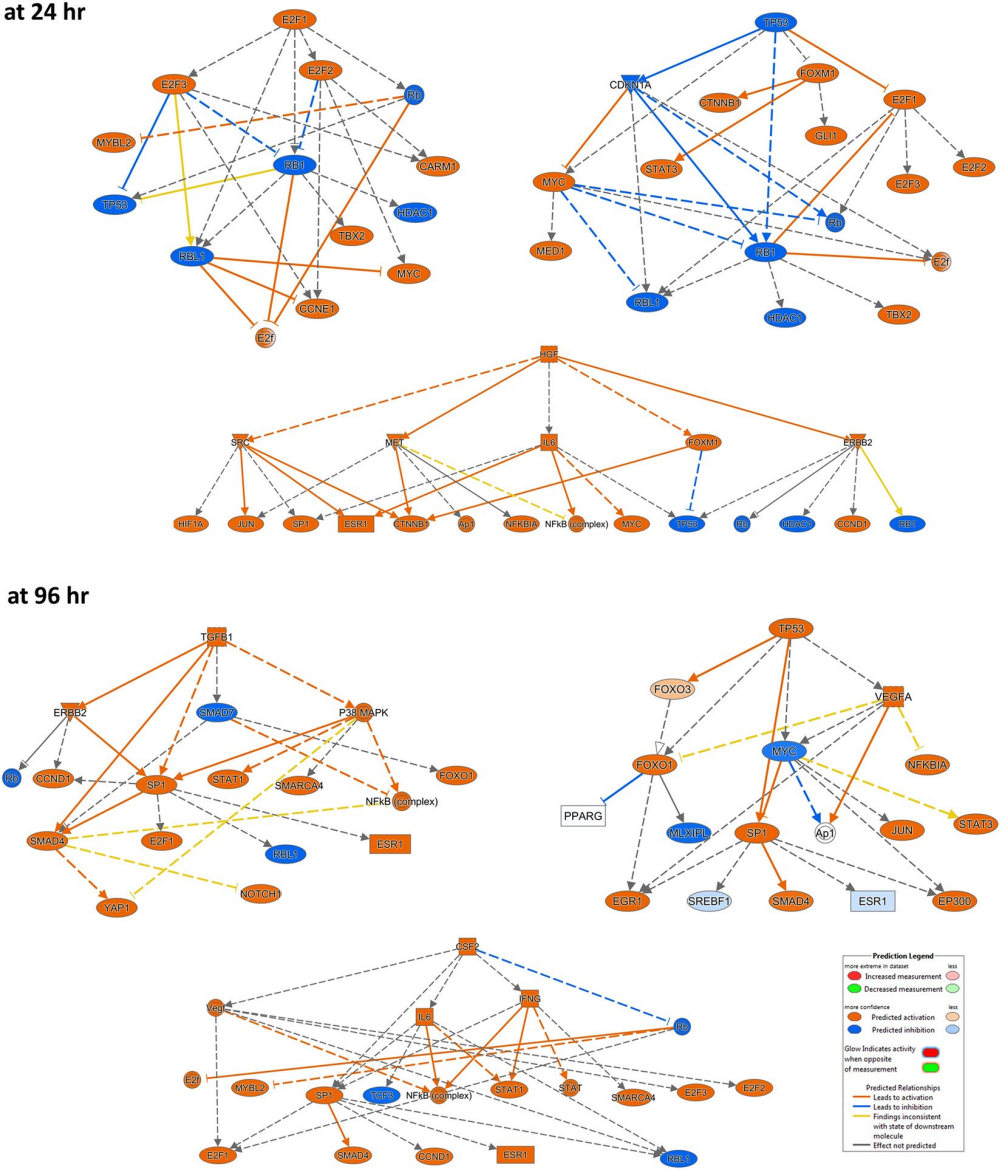
Mechanistic networks of upstream regulators and their predicted relationship by IPA®. The networks for top upstream regulators: E2F1, HGF and TP53 at 24 h and TGFB1, Vegf, and TP53 at 96 h are shown.

Activated regulators, such as *E2F1, CCND1, TBX2, RABL6, MYC, EP400, Vegf, FOXM1*, HGF, and *IL6*, and inhibited regulators, including *CDKN1A, NUPR1, Rb, HNF4A*, and *TP53*, may play important roles for early liver regeneration (Fig. 4). *TGFB, KRAS, IL1, ERK1/2* appears to be activated in ALLPS and PH (Fig. 4g and Supplementary Table S5). On the other hand, at 96 h, *CSF2, Cg, TGFB1, IFNG, IL5*, and *F2* were predicted to be activated in all procedures. *FOXM1, PTGER2, E2F3, TBX2, CCND1, E2F1*, and *Notch* were activated significantly in the ALPPS; *TNF, TP53, STAT3, PDGF*, and *IL1A* in the PH; *JUN* and *POU5F1* in the PVL. *Vegf, HGF, IL6, IL1B, NFKB*, *IFNA*, and *MITF* were activated both in ALPPS and PH (Fig. 4 and complete list in Supplementary Table S5). There were 101 and 23 shared regulators among the procedures at 24 h and 96 h, respectively (Fig. 4a and Supplementary Table S5). The upstream regulator analysis also revealed several activated mechanistic networks at early and late-stage of liver regeneration, including *E2F1, HGF* and *TP53* at 24 h and *TGFB1, Vegf*, and *TP53* at 96 h (Fig. 5).

## Discussion

We performed next-generation RNAseq approach to comprehensively delineate liver regeneration induced by ALPPS, PVL, and PH approaches at two time points (24 h and 96 h). Recently, we have replicated ALPPS in a rat model and presented its quick rejuvenation mode^17^. In this study, we identified pertinent common transcriptomic signatures at critical time points (i.e. early proliferation and late-proliferation phase) of liver regeneration and revealed the inter-procedural and temporal variations in gene expression patterns.

In response to injury, liver regeneration is achieved by the activation of otherwise functional, fully-differentiated hepatocytes as a result of the autocrine and paracrine signaling (e.g., cytokines and growth factors)^12,17^. Thus, major underlying changes in gene networks are expected to be seen at the cellular level. Depending on the initial injurious stimulus (or surgical procedure), such networks may have distinct elements as well as shared factors that are critical for regeneration. Therefore, delineation of the gene networks in liver regeneration is of particular interest. Moreover, understanding of signaling cascades and gene networks of the ALPPS, PVL and PH at 24 hours is vital as these may be critical factors for the initiation of regeneration or resulting in poor recovery, incomplete regeneration, and ultimately, liver failure.

Our data revealed that time-dependent factors appear to be the major source of variation in post-injury alterations of gene expression in liver regeneration. Early transcriptomic changes and the upstream regulators after all the procedures included cell cycle associated genes (*E2F1, CCND1, FOXM1, TP53*, and *RB1*), transcription factors (*Myc, E2F1, TBX2, FOXM1*)^23–28^, DNA replication regulators (*CDKN1A, EZH2, RRM2*)^29^, G1/S-transition regulators (*CCNB1, CCND1, RABL6*)^30^, cytokines and growth factors (*CSF2, IL-6, TNF, HGF, VEGF*, and *EGF*)^9,16,31,32^. At the cellular level, this corresponds to the transition from the quiescent G0 phase to active mitosis. Liver regeneration is governed by numerous growth factors, including HGF and EGF that are responsible priming the parenchymal and nonparenchymal liver cells and boosting their access into the cell cycle to proliferate to restore the original liver size after the surgical procedures^9,16,31,32^. Growth factors and signaling pathways activates cyclin-dependent kinases (CDKs), and upregulates the expression of *CCND1* that encodes cyclin D1. *CDK1* is essential for DNA replication and downstream formation of replication-initiation complexes in hepatocytes and shown to play a critical role in DNA replication control during rat liver regeneration following PH^33^. It is also required for the activity of *CCNB1*, a protein from cyclin regulatory proteins family that is essential for cell cycle control during G2/M (mitosis) transition.

Upstream events that can induce the quiescence-to-mitosis transition may potentially be important in liver regeneration, such as activation of *E2F1, CCND1, FOXM1*, and inhibition of *TP53*, and *RB1*. Notably, enhancer of zeste homolog 2 (*EZH2*) was among the early significantly up-regulated genes. As the functional subunit of Polycomb Repressive Complex 2 (*PRC2*), which normally regulates development and differentiation in healthy embryonic tissue, it is potentially involved in early de-differentiation that hepatocytes undergo to become highly proliferative^34^. Additionally, its up-regulation is consistent with the decreased lipid metabolism identified by our functional network analysis^35^. As a specialized function of hepatocytes, the decrease in lipid metabolism may well be an indication of dedifferentiation. Besides the critical inducers of dedifferentiation and G0-to-G1 transition, other molecules seem to be critical in sustaining a robust intracellular environment. One example is p400 E1A-associated protein (*EP400*), which was predicted to be an early upstream regulator. *EP400* is essential for the control of reactive oxygen species (ROS) intracellularly, maintaining an oxidative-stress free environment without which DNA damage, senescence, and apoptosis may ensue^36^. Another example is Lipocalin 2 (*LCN2*), an innate-immunity molecule with iron-sequestering properties. It has a wide expression in various tissues but has been particularly used as an early biomarker for kidney injury^37^. Recently, the increased expression level of *LCN2* has been demonstrated in acute liver injury as well^38–40^. Indeed, protein and mRNA levels of *Lcn2* is significantly increased after partial hepatectomy^41^.

Immune mediators of liver regeneration that showed a significant increase in expression immediately after the operations were *IL-6* and *VEGF*^17,42–44^. These molecules are known mediators of acute and chronic inflammatory response, carrying out essential functions in repairing tissue injury in all parts of the human body. *IL-6*, secreted by macrophages, is a known pyrogen and a potent stimulator of the acute phase reactants following infection or trauma. *VEGF* is secreted by endothelial cells to promote vasculogenesis and angiogenesis. In liver injury, *VEGF* mediates the proliferation and mobilization of liver sinusoidal endothelial cell progenitor cells form the bone marrow to allow for the formation of new sinusoids in the regenerating, highly-vascular liver tissue^45^.

The network analysis of up-regulated genes at 96 h post-operatively revealed an expression profile predominantly in mediating tissue reconstruction, including cellular movement, system development, extracellular matrix organization and restoring specialized hepatocytic functions (e.g., carbohydrate transport and morphogenesis). Of note, this phase seems to be orchestrated by the macrophages (known as Kuppfer cells in the liver). Transforming growth factor-β *(TGF-β)*, a potent inhibitor of inflammation and a stimulator of healing and tissue repair that is secreted by macrophages, was significantly up-regulated at 96 h. Furthermore, the analysis also highlighted genes such as *LGALS3, FCGR2A, SPARC, integrin*, and *collagens*, that may play significant roles in tissue regeneration and repair^46–48^. These genes closely coordinate their function with *TGF-β* in tissue organization, matrix structure and cell-to-cell interactions. The knockout mice of secreted protein acidic and rich in cysteine (*SPARC*) showed a reduction of expression of *TGF-β1* and collagen in hepatic tissue^49^.

Among the three examined procedures, as noted in the clustering and upstream regular analyses, ALPPS and PH shared many significantly regulated genes whose expression were not otherwise significantly changed in PVL, especially at the early phase of regeneration. One possible explanation lies in the differences of the type of injury caused by each procedure. Specifically, ALPPS and PH both require the removal of hepatic parenchyma. On the other hand, the main stimulant of regeneration in PVL is ischemia and oxygen deprivation. Clinically, PVL is the least invasive and has the lowest rate of morbidity and mortality but also has a slower rate of regeneration than the other procedures^50^. Compared to PVL, the upstream regulator analysis revealed the activation of *TGF-β, KRAS, ERK1/2, IL1*, and *INS* at early phase and *Vegf, HGF, Interferan alpha, IL6*, *IL1B*, and *NFKB* at the later regeneration phase after ALPPS and PH. On the other hand, compared to PH and PVL, the cell cycle associated genes, cytokines, and transcription regulators, such as *E2F1, TBX2, FOXM1*, and *EP400*, are still activated after ALPPS at later regeneration phase. Finally, the Notch signaling, which is a complex signaling pathway that is crucial for the development of multiple organs, was also seemed to be activated in ALPPS. In the liver, it controls the hepatic cell differentiation into, and the formation of, the biliary system^51^. In addition, notch proteins have an extracellular membrane component with epidermal growth-like factor (*EGF*) repeats, which may be responsible for the high growth rate seen clinically in ALPPS^52^.

The upstream analyses also indicated the predicted inhibition of *NUPR1, CDKN2A, Rb, PAX6*, and *TP53*, at initial phase of liver regeneration^53,54^. However, at the later stage of liver regeneration, *TP53* is activated, especially after PH. Furthermore, the mechanistic network of upstream regulators and their predicted relationship based on the observed gene expression changes in our data reveals the working mechanism of TP53 at early and late stage of the liver regeneration, as demonstrated in Fig. 5. p53 regulates liver homeostasis, and initiation of cell proliferation through proliferative signaling and disruption of p53 signaling lead to faster recovery^53,54^.

The limitation of the study is that we examined the liver proliferation at two time points, early phase (i.e. 24 h) and late-stage (i.e. 96 h). Our earlier study of ALPPS and PVL indicated higher proliferation index (PI) at 24 h and 48 hr comparing to 96 h, and there was no significant difference between the two time-points (i.e. 24 h vs. 48 h)^17^. The future remnant liver volume (FRLV) ratio was significantly higher in ALPPS comparing to PVL at 24 hr and 96 hr time points, but not significantly different at 48 h or 1 week. Higher FRL ratio is critical factor in improving surgical outcomes and liver regeneration. In addition, the ALPPS model has significantly more inflammatory cells infiltration at 24 hr comparing to 48 hr; higher infiltration of inflammatory might promoted earlier liver regeneration in the ALPPS model. It also indicated higher portal pressure in ALPPS group at 24 hr comparing to other time points; having higher portal pressure might act as physical stressor that contribute to ignite the regeneration process. Xu *et al*. studied the expressed genes in regenerating rat liver after PH, also reported that temporal patterns of gene expression were similar at 48 h and 96 h after PH^55^. Nevertheless, more time points should worth to be assessed to fully investigate the liver proliferation process. While recognizing this limitation, we believe we have largely achieved our aim, as the major objective of the study is to fish out early phase (i.e. 24 h) and late-stage (i.e. 96 h) liver regeneration molecular markers and examining molecular differences between these phases in the ALPSS, PVL and PH models that have provided unique molecular signatures.

In summary, our study presents a comprehensive transcriptomic profiling of three surgical procedures that are commonly used in clinical practice and identified the inter-procedural and temporal variations in gene expression patterns in each surgical procedure. Identification of molecular signatures and signaling pathways specific to each surgical procedure further our understanding of key regulators of liver regeneration as well as patient populations that are likely to benefit from each procedure.

## Supplementary information

Supplementary Information.

Supp Table S1.

Supp Table S2.

Supp Table S3.

Supp Table S4.

Supp Table S5.

## Data Availability

The datasets generated and analyzed during the current study is available at this link (https://www.dropbox.com/s/hgewpv07qce6fed/RNASeqTopHataligned_Normdata_sample29.txt?dl=0).

## References

[1] Kimura N (2012). Gene expression of ATP-binding cassette transporters during liver regeneration after 90% hepatectomy in rats. International journal of molecular medicine.

[2] Fukuhara Y (2003). Gene expression profile in the regenerating rat liver after partial hepatectomy. Journal of hepatology.

[3] Colak D (2010). Integrative and comparative genomics analysis of early hepatocellular carcinoma differentiated from liver regeneration in young and old. Mol Cancer.

[4] Lu X (2016). Integrated analysis of microRNA and mRNA expression profiles highlights the complex and dynamic behavior of toosendanin-induced liver injury in mice. Sci Rep.

[5] Chen XG, Xu CS, Liu YM (2013). Involvement of ERK1/2 signaling in proliferation of eight liver cell types during hepatic regeneration in rats. Genetics and molecular research: GMR.

[6] Hadden WJ (2016). Resection of colorectal liver metastases and extra-hepatic disease: a systematic review and proportional meta-analysis of survival outcomes. HPB: The Official Journal of the International Hepato Pancreato Biliary Association.

[7] Schnitzbauer AA (2012). Right portal vein ligation combined with *in situ* splitting induces rapid left lateral liver lobe hypertrophy enabling 2-staged extended right hepatic resection in small-for-size settings. Annals of surgery.

[8] Locker J (2003). A common set of immediate-early response genes in liver regeneration and hyperplasia. Hepatology (Baltimore, Md.).

[9] Dhar DK, Mohammad GH, Vyas S, Broering DC, Malago M (2015). A novel rat model of liver regeneration: possible role of cytokine induced neutrophil chemoattractant-1 in augmented liver regeneration. Annals of Surgical Innovation and Research.

[10] Schlegel Andrea, Lesurtel Mickael, Melloul Emmanuel, Limani Perparim, Tschuor Christoph, Graf Rolf, Humar Bostjan, Clavien Pierre A. (2014). ALPPS. Annals of Surgery.

[11] Blanpain C., Fuchs E. (2014). Plasticity of epithelial stem cells in tissue regeneration. Science.

[12] Font-Burgada J (2015). Hybrid Periportal Hepatocytes Regenerate the Injured Liver without Giving Rise to Cancer. Cell.

[13] Bhate A (2015). ESRP2 controls an adult splicing programme in hepatocytes to support postnatal liver maturation. Nature communications.

[14] Xu CS (2004). Gene expression differences of regenerating rat liver in a short interval successive partial hepatectomy. World journal of gastroenterology.

[15] Nagano Y (2004). Gene expression profile analysis of regenerating liver after portal vein ligation in rats by a cDNA microarray system. Liver international: official journal of the International Association for the Study of the Liver.

[16] Garcia-Perez R (2015). Associated Liver Partition and Portal Vein Ligation (ALPPS) vs Selective Portal Vein Ligation (PVL) for Staged Hepatectomy in a Rat Model. Similar Regenerative Response?. PloS one.

[17] Dhar DK, Mohammad GH, Vyas S, Broering DC, Malago M (2015). A novel rat model of liver regeneration: possible role of cytokine induced neutrophil chemoattractant-1 in augmented liver regeneration. Annals of surgical innovation and research.

[18] Langmead B, Trapnell C, Pop M, Salzberg SL (2009). Ultrafast and memory-efficient alignment of short DNA sequences to the human genome. Genome biology.

[19] Trapnell C (2012). Differential gene and transcript expression analysis of RNA-seq experiments with TopHat and Cufflinks. Nature protocols.

[20] Anders S, Huber W (2010). Differential expression analysis for sequence count data. Genome biology.

[21] Dennis G (2003). DAVID: Database for Annotation, Visualization, and Integrated Discovery. Genome biology.

[22] Xia J, Gill EE, Hancock RE (2015). NetworkAnalyst for statistical, visual and network-based meta-analysis of gene expression data. Nature protocols.

[23] Farra R (2015). Impairment of the Pin1/E2F1 axis in the anti-proliferative effect of bortezomib in hepatocellular carcinoma cells. Biochimie.

[24] Ella E (2014). Specific genomic and transcriptomic aberrations in tumors induced by partial hepatectomy of a chronically inflamed murine liver. Oncotarget.

[25] Wang HB (2018). Myc and ChREBP transcription factors cooperatively regulate normal and neoplastic hepatocyte proliferation in mice. J Biol Chem.

[26] Berthet B, DiCostanzo J, Di Costanzo V, Frigerio JM, Dagorn JC (1998). Expression of genes associated with liver regeneration following partial hepatectomy in the rat. Influence of Epidermal Growth Factor and Interleukin 6. Ann Gastroent Hepato.

[27] Yin L, Wang YH, Guo XQ, Xu CS, Yu GY (2018). Comparison of gene expression in liver regeneration and hepatocellular carcinoma formation. Cancer Manag Res.

[28] Chishti MA (2013). Induction of cell proliferation in old rat liver can reset certain gene expression levels characteristic of old liver to those associated with young liver. Age.

[29] Yin L, Wang YY, Lin YZ, Yu GY, Xia QF (2019). Explorative analysis of the gene expression profile during liver regeneration of mouse: a microarray-based study. Artif Cell Nanomed B.

[30] Shizu R (2016). PXR stimulates growth factor-mediated hepatocyte proliferation by cross-talk with the FOXO transcription factor. Biochem J.

[31] Shi H (2015). A preliminary study of ALPPS procedure in a rat model. Scientific reports.

[32] Yao LB (2018). *In situ* splitting after selective partial portal vein ligation or simultaneous hepatic artery ligation promotes liver regeneration. Sci Rep.

[33] Garnier D, Loyer P, Ribault C, Guguen-Guillouzo C, Corlu A (2009). Cyclin-dependent kinase 1 plays a critical role in DNA replication control during rat liver regeneration. Hepatology (Baltimore, Md.).

[34] Bae WK (2015). The methyltransferases enhancer of zeste homolog (EZH) 1 and EZH2 control hepatocyte homeostasis and regeneration. FASEB journal: official publication of the Federation of American Societies for Experimental Biology.

[35] Vella S (2013). EZH2 down-regulation exacerbates lipid accumulation and inflammation in *in vitro* and *in vivo* NAFLD. International journal of molecular sciences.

[36] Mattera L (2010). The E1A-associated p400 protein modulates cell fate decisions by the regulation of ROS homeostasis. PLoS genetics.

[37] Bolignano D (2008). Neutrophil gelatinase-associated lipocalin (NGAL) as a marker of kidney damage. American journal of kidney diseases: the official journal of the National Kidney Foundation.

[38] Borkham-Kamphorst E (2013). Protective effects of lipocalin-2 (LCN2) in acute liver injury suggest a novel function in liver homeostasis. Biochimica et biophysica acta.

[39] Cheng Q (2014). The roles of lipocalin-2 in small-for-size fatty liver graft injury. Annals of surgery.

[40] Roudkenar MH (2008). Gene expression profiles in mouse liver cells after exposure to different types of radiation. J Radiat Res.

[41] Lai HS, Wu YM, Lai SL, Lin WH (2013). Lipocalin-2 gene expression during liver regeneration after partial hepatectomy in rats. International journal of surgery.

[42] Chinnici CM (2019). Mesenchymal stromal cells isolated from human fetal liver release soluble factors with a potential role in liver tissue repair. Differentiation.

[43] Gu K (2011). Hepatic Regeneration after Sublethal Partial Liver Irradiation in Cirrhotic Rats. J Radiat Res.

[44] Jia CK (2011). Advances in the regulation of liver regeneration. Expert Rev Gastroent.

[45] Wang L (2012). Hepatic vascular endothelial growth factor regulates recruitment of rat liver sinusoidal endothelial cell progenitor cells. Gastroenterology.

[46] Li XW (2010). Proteomics analysis of plasma membrane from liver sinusoidal endothelial cells after partial hepatectomy by an improved two-dimensional electrophoresis. Mol Cell Biochem.

[47] Issa Razao, Zhou Xiaoying, Trim Nathan, Millward‐Sadler Harry, Krane Stephen, Benyon Christopher, Iredale John (2002). Mutation in collagen‐I that confers resistance to the action of collagenase results in failure of recovery from CCl 4 ‐induced liver fibrosis, persistence of activated hepatic stellate cells, and diminished hepatocyte regeneration. The FASEB Journal.

[48] Li J (2014). Human Hepatic Progenitor Cells Express Hematopoietic Cell Markers CD45 and CD109. Int J Med Sci.

[49] Atorrasagasti C (2013). Lack of the Matricellular Protein SPARC (Secreted Protein, Acidic and Rich in Cysteine) Attenuates Liver Fibrogenesis in Mice. PloS one.

[50] Treska V (2016). Methods to Increase Future Liver Remnant Volume in Patients with Primarily Unresectable Colorectal Liver Metastases: Current State and Future Perspectives. Anticancer research.

[51] Morell CM, Strazzabosco M (2014). Notch signaling and new therapeutic options in liver disease. Journal of hepatology.

[52] Kopan Raphael, Ilagan Ma. Xenia G. (2009). The Canonical Notch Signaling Pathway: Unfolding the Activation Mechanism. Cell.

[53] Krstic Jelena, Galhuber Markus, Schulz Tim, Schupp Michael, Prokesch Andreas (2018). p53 as a Dichotomous Regulator of Liver Disease: The Dose Makes the Medicine. International Journal of Molecular Sciences.

[54] Borude P (2018). Pleiotropic Role of p53 in Injury and Liver Regeneration after Acetaminophen Overdose. The American journal of pathology.

[55] Xu CS (2005). Expressed genes in regenerating rat liver after partial hepatectomy. World journal of gastroenterology.

