# Publisher Correction: RNA-Seq transcriptome profiling in three liver regeneration models in rats: comparative analysis of partial hepatectomy, ALLPS, and PVL

**DOI:** 10.1038/s41598-020-64447-w

**Published:** 2020-05-05

**Authors:** Dilek Colak, Olfat Al-Harazi, Osama M. Mustafa, Fanwei Meng, Abdullah M. Assiri, Dipok K. Dhar, Dieter C. Broering

**Affiliations:** 1 Biostatistics, Epidemiology, and Scientific Computing Department, King Faisal Specialist Hospital and Research Center, Riyadh, Saudi Arabia; 2 Department of Surgery and Organ Transplantation Center, King Faisal Specialist Hospital and Research Center, Riyadh, Saudi Arabia; 3 Comparative Medicine Department, King Faisal Specialist Hospital and Research Center, Riyadh, Saudi Arabia; 4 Institute for Research and Medical Consultations, Imam Abdulrahman Bin Faisal University, Dammam, Saudi Arabia; 5 College of Medicine, AlFaisal University, Riyadh, Saudi Arabia; 6 Institute for Liver and Digestive Health, University College London, Royal Free Hospital, London, UK

Correction to: *Scientific Reports* 10.1038/s41598-020-61826-1, published online 23 March 2020

In the original PDF version of this Article, Dilek Colak was omitted as jointly supervising this work.

This error has now been corrected in the PDF version of the paper; the HTML version was correct from the time of publication.

