# Supplementary material for: RNA-Seq transcriptome profiling in three liver regeneration models in rats: comparative analysis of partial hepatectomy, ALLPS, and PVL
Source: Sci Rep. 2020 Mar 23;10:5213. doi: 10.1038/s41598-020-61826-1 (PMC7089998; doi:10.1038/s41598-020-61826-1)
Supplement: Supplementary file 1 — Supplementary Information. [file 41598_2020_61826_MOESM1_ESM.docx]

**RNA-Seq transcriptome profiling in three liver regeneration models in rats: comparative analysis of partial hepatectomy, ALLPS, and PVL**

Dilek Colak^1*^, Olfat Al-Harazi^1^, Osama M. Mustafa^1^, Fanwei Meng^2,3^, Abdullah M. Assiri^3,4,5^, Dipok K. Dhar^2,3,6*^, Dieter C. Broering^2,5^

^1^ Biostatistics, Epidemiology, and Scientific Computing Department, King Faisal Specialist Hospital and Research Center, Riyadh, Saudi Arabia.

^2^ Department of Surgery and Organ Transplantation Center, King Faisal Specialist Hospital and Research Center, Riyadh, Saudi Arabia.

^3^ Comparative Medicine Department, King Faisal Specialist Hospital and Research Center, Riyadh, Saudi Arabia.

^4^ Institute for Research and Medical Consultations, Imam Abdulrahman Bin Faisal University, Dammam, Saudi Arabia.

^5^ College of Medicine, AlFaisal University, Riyadh, Saudi Arabia.

^6^ Institute for Liver and Digestive Health, Regenerative Medicine & Fibrosis Group University College London, Royal Free Hospital, London, UK

*Correspondence and requests for materials should be addressed to D.C. (email: [dkcolak@gmail.com](mailto:dkcolak@gmail.com)) or D.K.D. (email: [ddhar20@kfshrc.edu.sa](mailto:ddhar20@kfshrc.edu.sa))

**Supplementary Information**

**
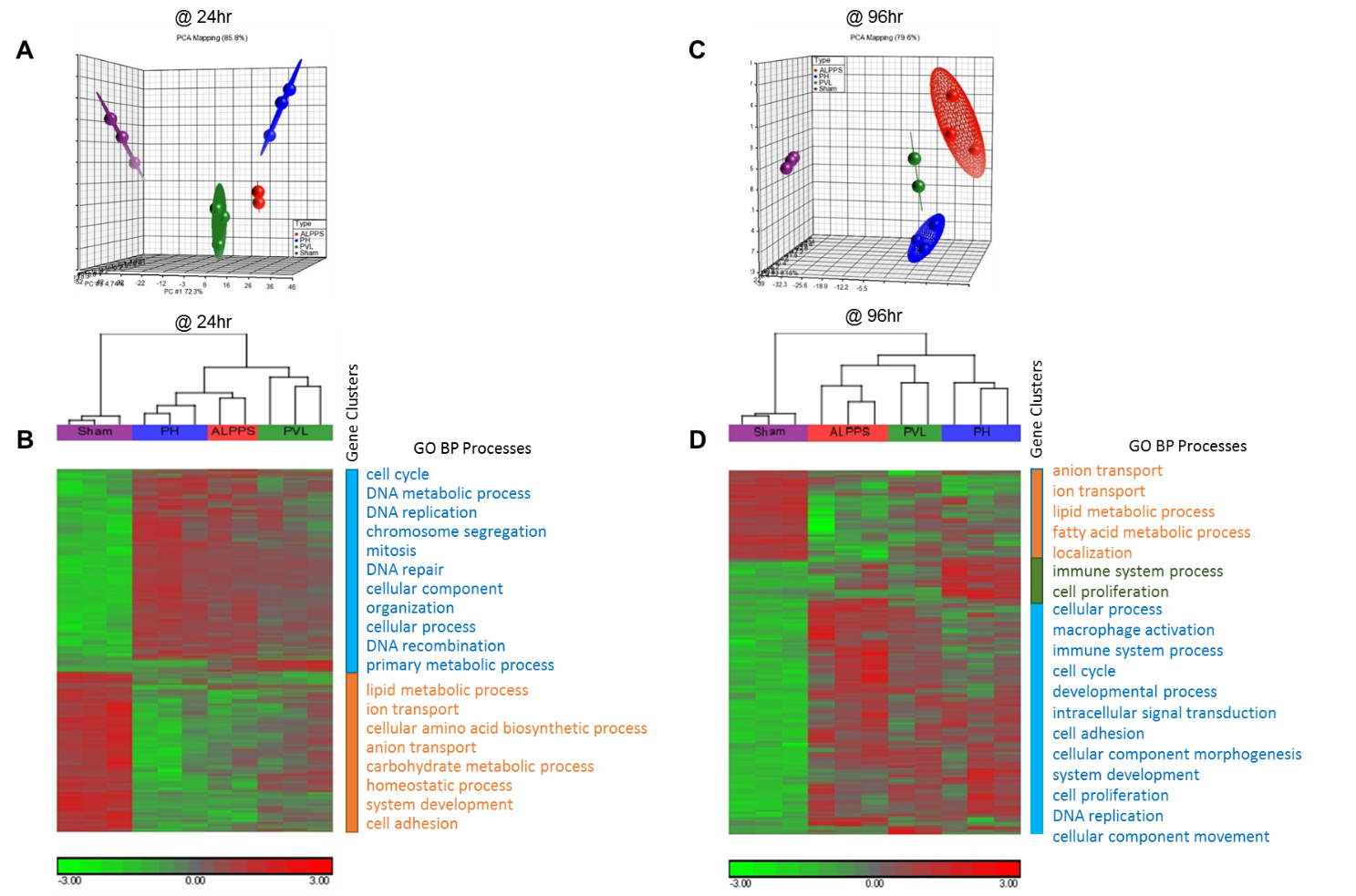
**

**Supplementary Figure S1. (A, C)** Unsupervised PCA analysis separates samples according to the surgical types at 24h and 96h, respectively. Different colors indicate different surgical types. **(B, D)** Hierarchical clustering of genes that are significant in at least one surgical procedure (with respect to sham group) at 24h (left panel) and 96h (right panel), respectively. Red and green denote highly and weakly expressed genes, respectively. Next to the heatmaps, the figures display the most associated GO biological processes for each cluster of genes.


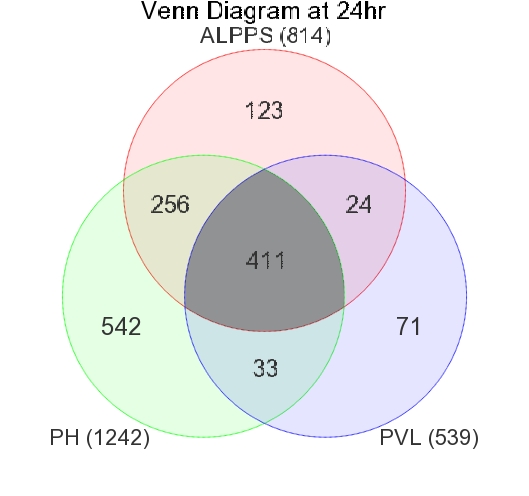

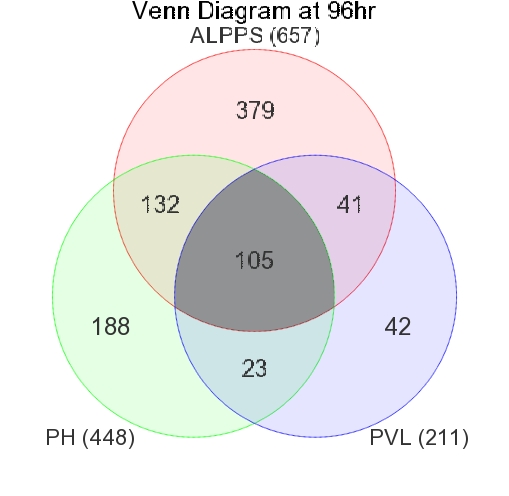


A

@ 96hr

@ 24hr

**
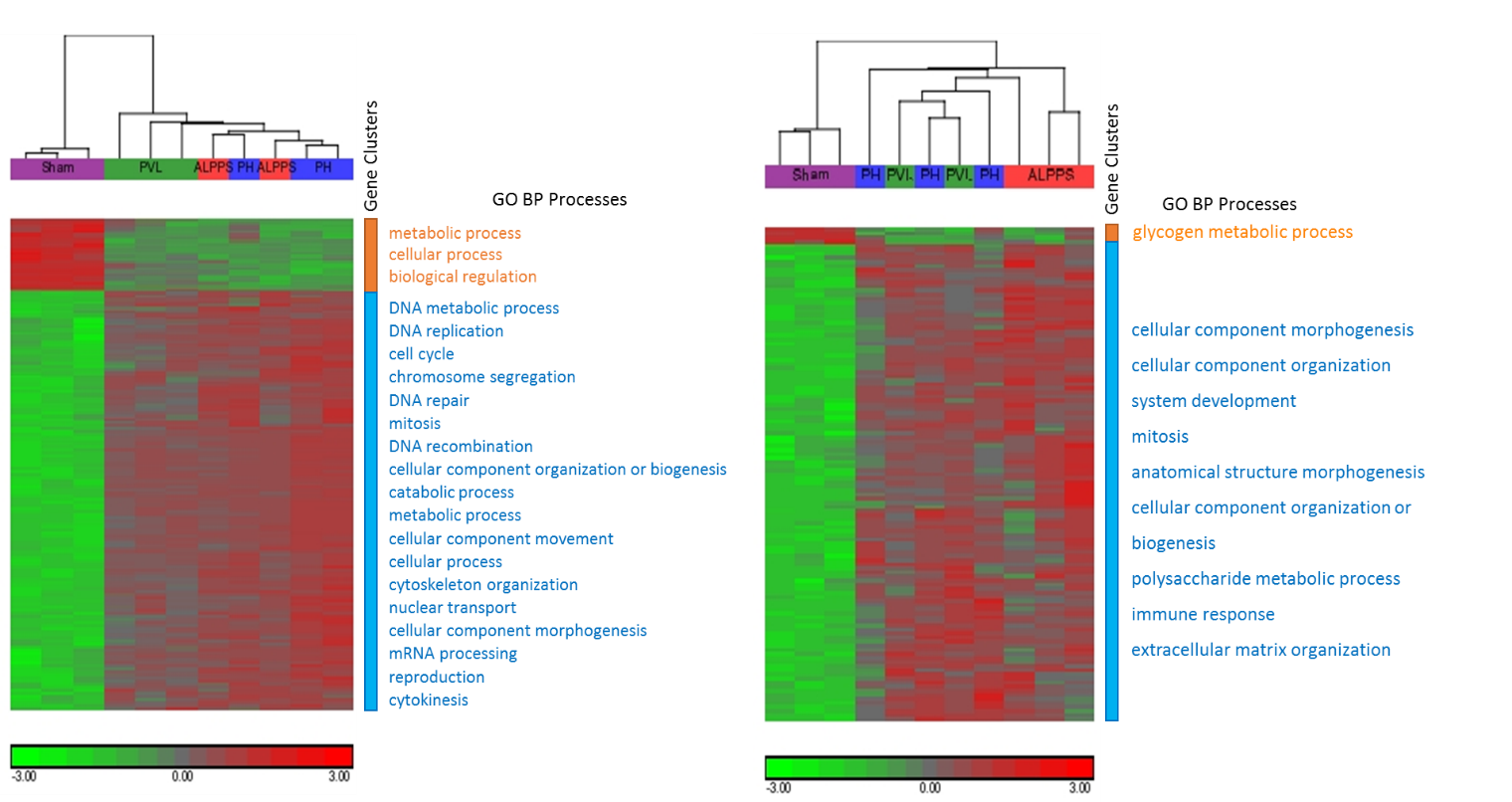
**

B

**Supplementary Figure S2. (A)** Venn diagrams representing the differentially expressed genes that are commonly expressed in ALPPS, PH and PVL at 24h (left panel) and 96h (right panel), respectively. **(B)** Heatmaps of genes that are significantly expressed in ALPPS, PH and PVL procedures at 24h (left panel) and 96h (right panel) The figure shows the most associated GO biological processes for each cluster of genes. Red and green denote highly and weakly expressed genes, respectively.

**
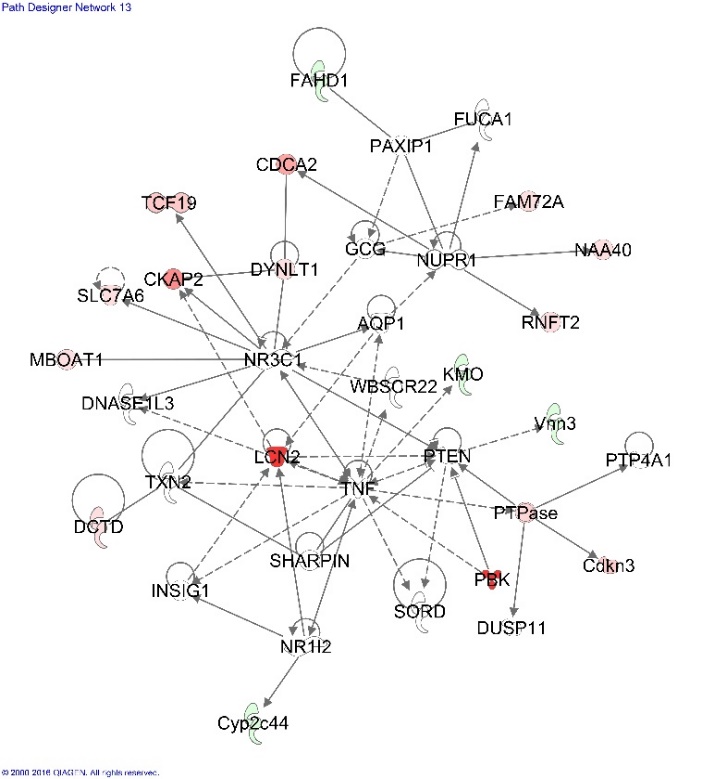

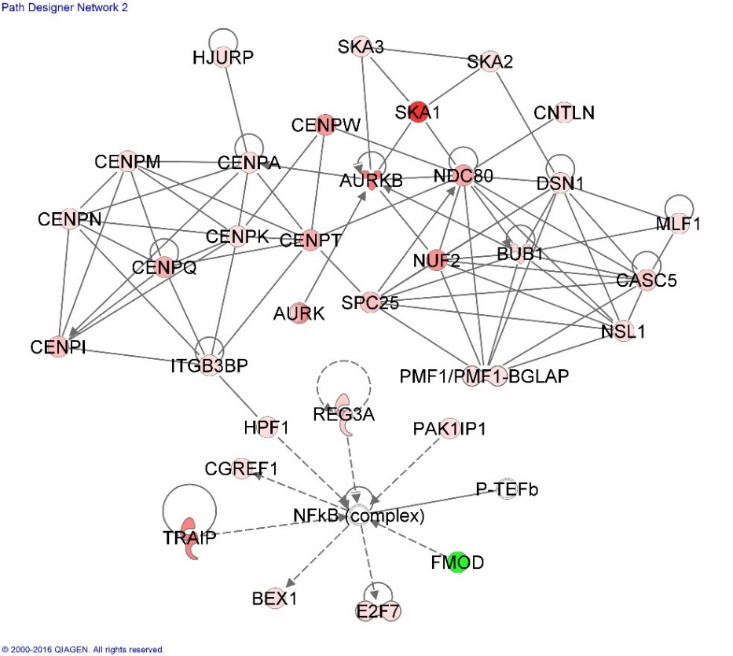

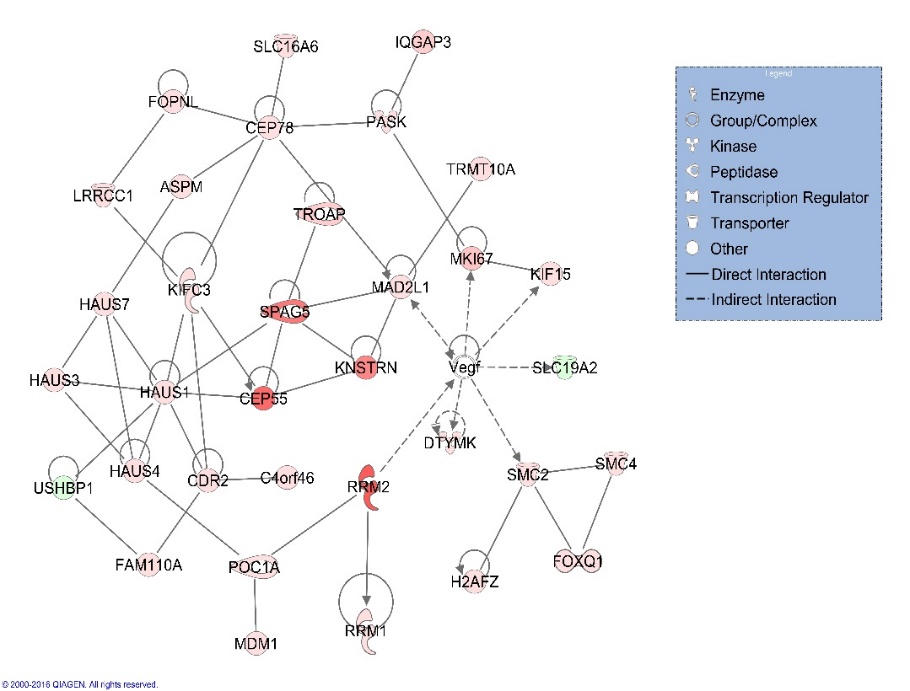
**

**
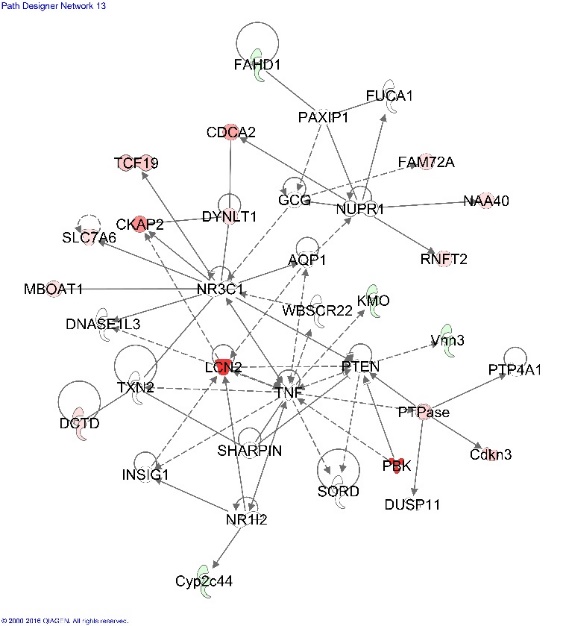
**

**Supplementary Figure S3.** Top three significant subnetworks for DEGs at 24h post-operation that were commonly regulated among ALPPS, PH and PVL. The subnetworks were generated through the use of IPA (QIAGEN Inc., https://www.qiagenbioinformatics.com/products/ingenuity-pathway-analysis). Green indicates down-regulated, and red, up-regulated. The color intensity is correlated with fold change. Straight and dashed lines represent direct or indirect gene to gene interactions, respectively.


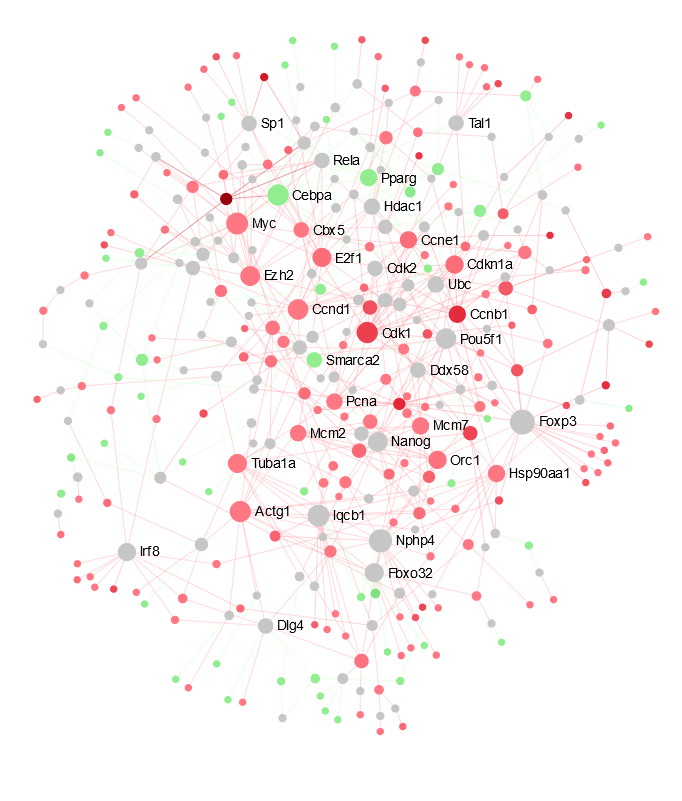
 **A) ALLPS**

**24hr**

**
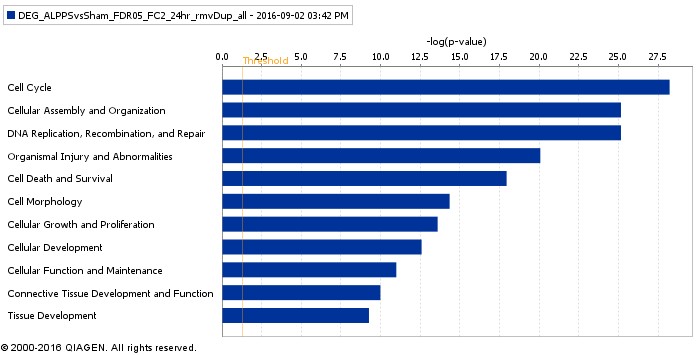
**

**
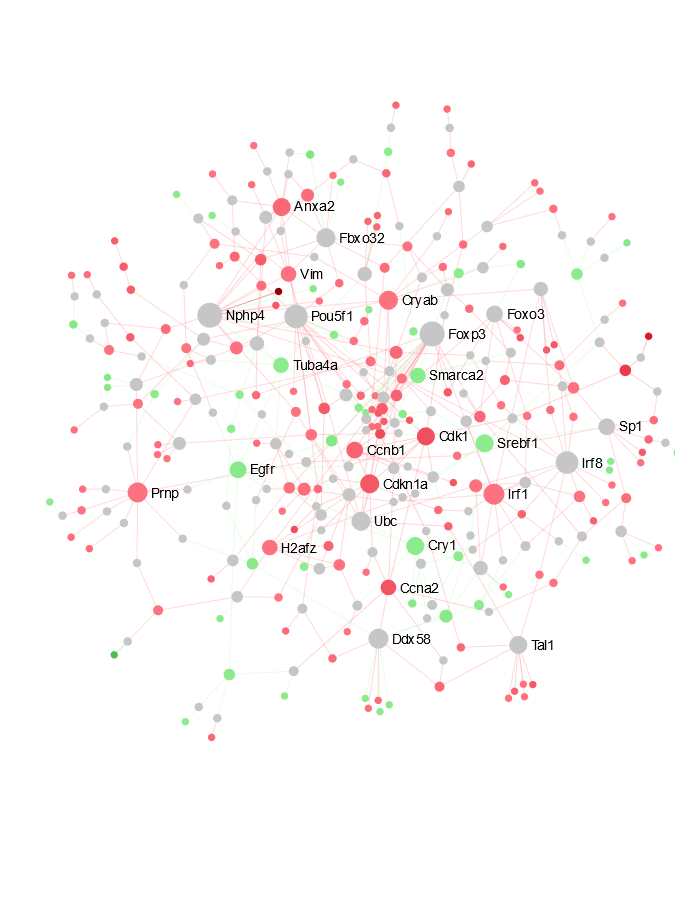

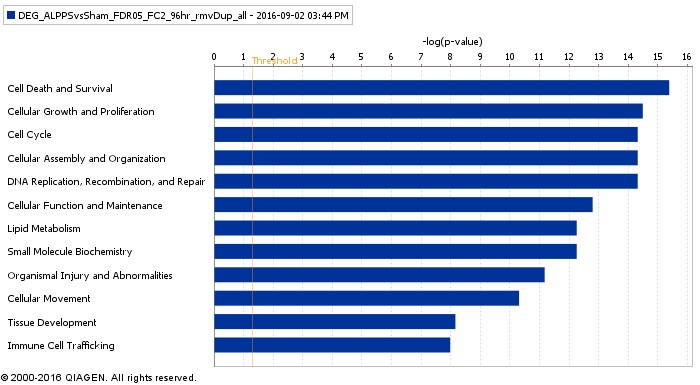
**

**96hr**

**B) PVL**

**24hr**


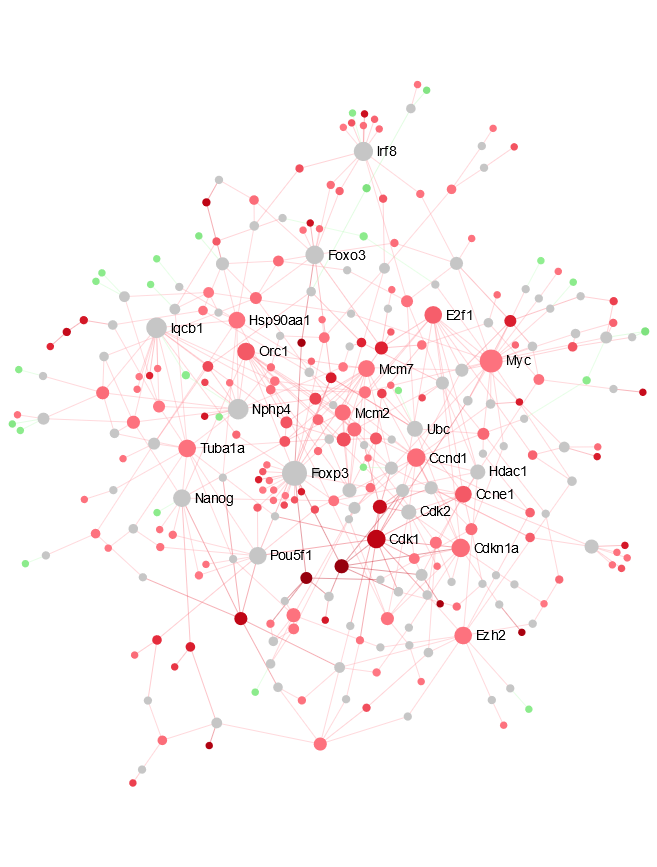

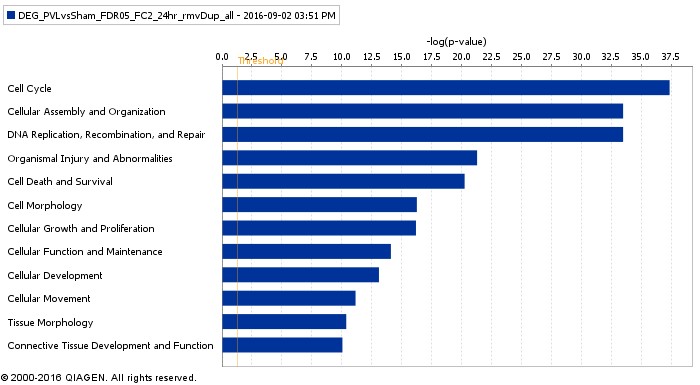


**96hr**


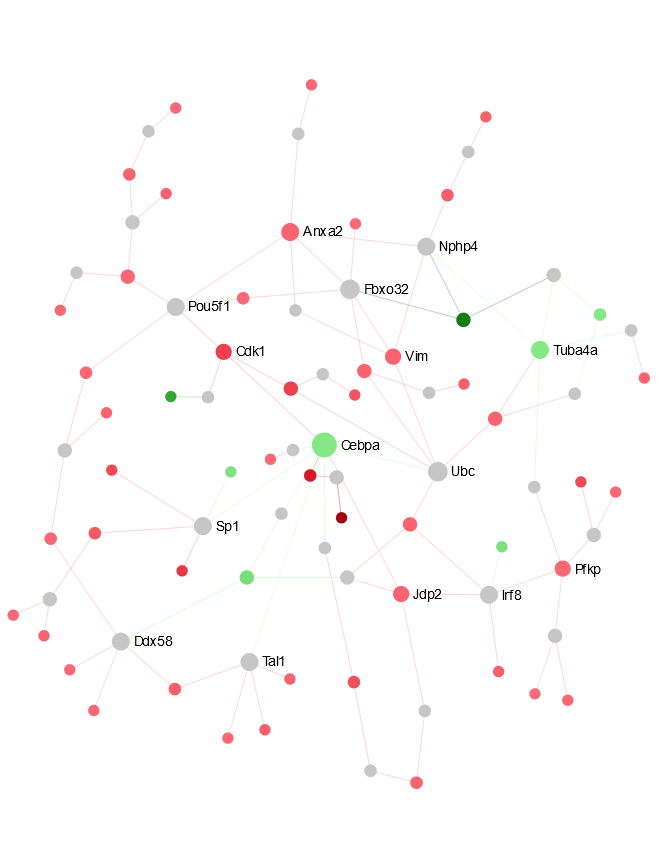

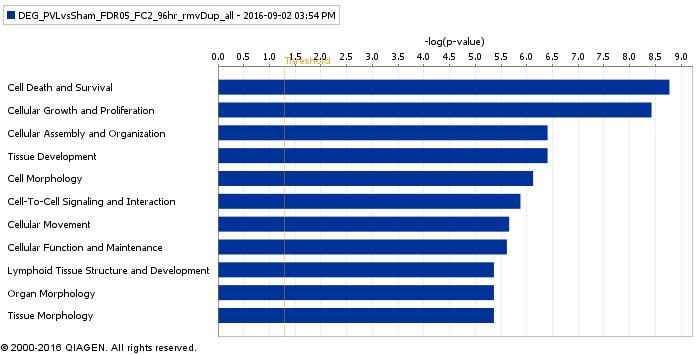


**C) PH**

**24hr**


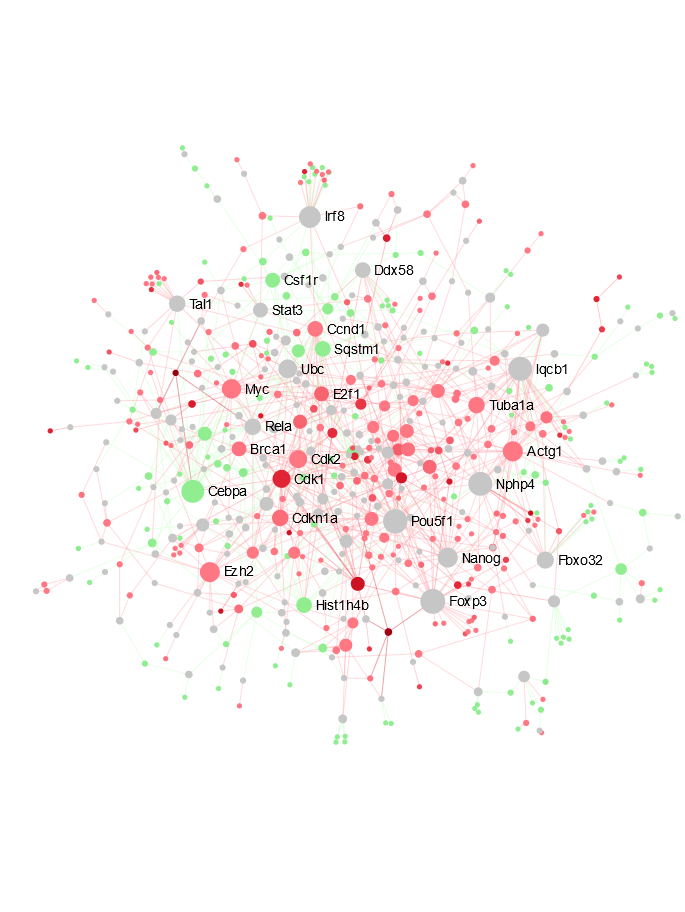

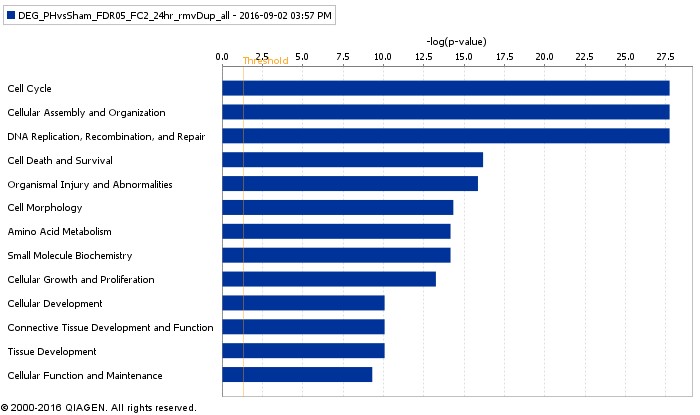

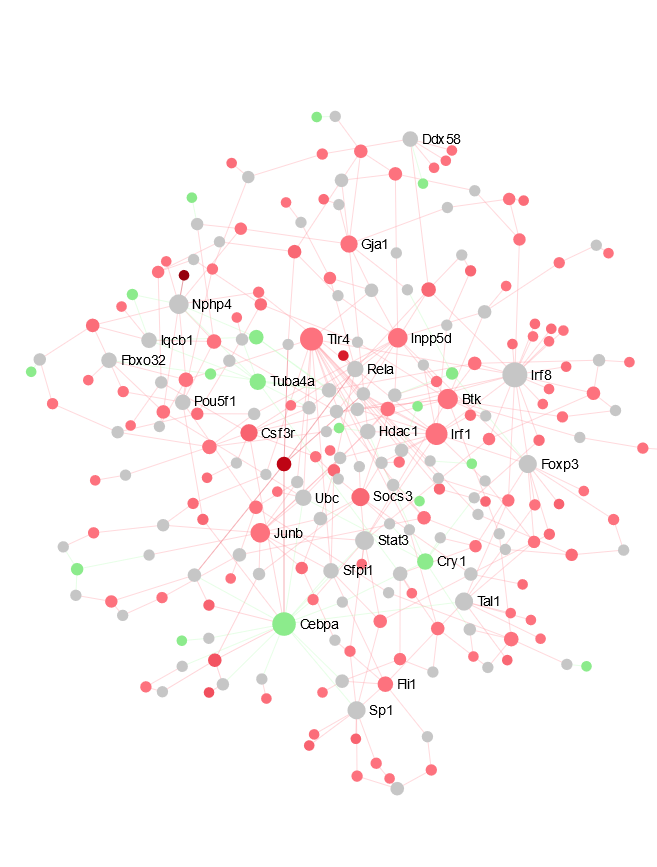

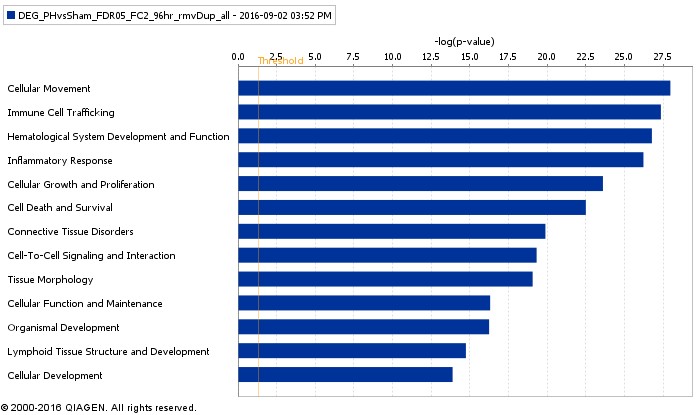


**96hr**

**Supplementary Figure S4.** Gene networks and functional analyses of DEGs for each surgical procedure at 24h and 96h post-operation after ALPPS **(A)**, PVL **(B)** and PH **(C)**, respectively with the hub genes labelled (right panel). Red and green denote up- and down-regulated genes, respectively, and grey indicates direct interactors of the DEGs. The color intensity is correlated with fold change. The bar charts (left panel) represent the gene ontology and functional analyses of DEGs in three surgical groups. X-axis indicates the significance (–log10(p-value)) of the functional association. The threshold line represents a P value of 0.05. Data were analyzed through the use of Network Analyst and IPA (QIAGEN Inc., <https://www.qiagenbioinformatics.com/products/ingenuitypathway-analysis>).
